# Evolution of the parasitic wasp subfamily Rogadinae (Braconidae): phylogeny and evolution of lepidopteran host ranges and mummy characteristics

**DOI:** 10.1186/1471-2148-8-329

**Published:** 2008-12-04

**Authors:** Alejandro Zaldívar-Riverón, Mark R Shaw, Alberto G Sáez, Miharu Mori, Sergey A Belokoblylskij, Scott R Shaw, Donald LJ Quicke

**Affiliations:** 1 Departamento de Biodiversidad y Biología Evolutiva, Museo Nacional de Ciencias Naturales (CSIC), c/José Gutierrez Abascal 2, 28006, Madrid, Spain; 2 Honorary Research Associate, National Museums of Scotland, Chambers Street, Edinburgh, EH1 1JF, UK; 3 Division of Biology and Centre for Population Biology, Imperial College London, Silwood Park Campus, Ascot, Berkshire, SL5 7PY, UK; 4 Department of Entomology, The Natural History Museum, London, SW7 5BD, UK; 5 Zoological Institute, Russian Academy of Sciences, Universitetskaya nab. 1, St. Petersburg 199034, Russia; 6 Museum and Institute of Zoology PAN, Wilcza 64, Warsaw 00-679, Poland; 7 University of Wyoming Insect Museum, Department of Renewable Resources, University of Wyoming, Laramie, WY, 82071-3354, USA

## Abstract

**Background:**

The braconid subfamily Rogadinae is a large, cosmopolitan group of endoparasitoid wasps characterised by 'mummifying' their lepidopteran host larvae, from which the adult subsequently emerges. Rogadines attack a variety of both macro- and microlepidopteran taxa, although the speciose genus *Aleiodes *almost exclusively attacks macrolepidopterans. Here, we investigate the phylogenetic history of the Rogadinae, revise their higher-level classification and assess the evolution of their host ranges and mummy types. We also assess the divergence times within the subfamily and discuss the reasons for the extraordinary evolutionary diversification of *Aleiodes*.

**Results:**

Our Bayesian analyses weakly support the monophyly of the subfamily. A clade comprising all *Aleiodes *species and some other taxa is not nested within the tribe Rogadini as previously supposed, but instead is recovered as sister to the Yeliconini, with the remaining Rogadini genera being recovered as sister to the Stiropiini. The Rogadinae is estimated to have originated during the mid to late Eocene, 36.1–51.62 MYA. Molecular dating gives a more recent origin for the *Aleiodes *clade (17.98–41.76 MYA) compared to the origins proposed for two of its principal lepidopteran host groups (Noctuidae: 60.7–113.4 MYA; Geometridae 48–62 MYA). The Bayesian ancestral reconstruction of the emergence habits from the mummified hosts weakly recovered an anterior emergence as the ancestral condition for the subfamily. Producing a hard mummy has evolved at various times independently, though most of the species with this biology belong to the *Aleiodes *clade.

**Conclusion:**

Based on our results, we erect the tribe Aleiodini **nov. **to include *Aleiodes *and *Heterogamus ***stat. rev. ***Cordylorhogas*, *Pholichora *and *Hemigyroneuron *are synonymised with *Aleiodes*. The molecular dating of clades and the ancestral reconstruction of host ranges support the hypothesis that radiation within *Aleiodes s. s. *was due to host recruitment leading to host range expansion followed by speciation, and not to parasitoid-host coevolution. Within the Rogadinae, variation in the site of emergence from the mummified host probably evolved as a consequence of the mummy's site and mode of formation, and the extent of mummy tanning/hardness to the degree of protection needed in relation to the cost of providing it.

## Background

**Figure 1:**
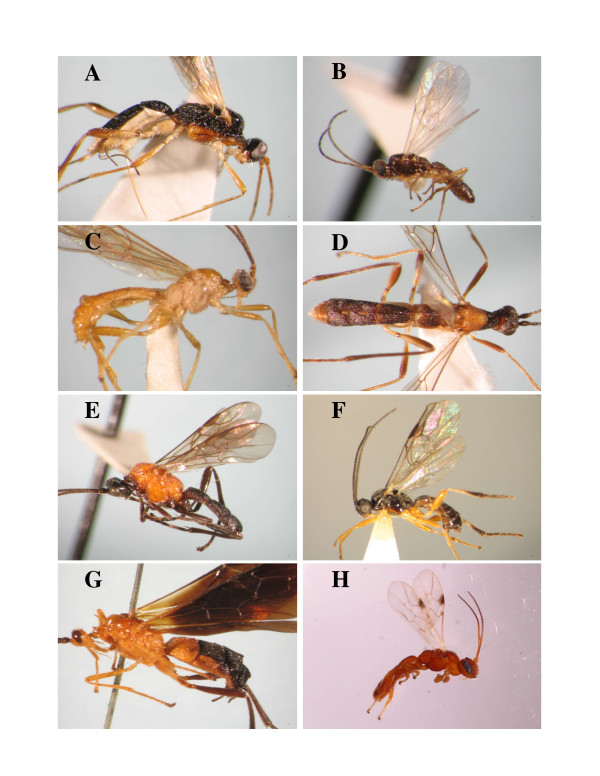
**Photographs of Rogadinae wasps**. A. Clinocentrini: *Clinocentrus *sp. B. Stiropiini: *Stiropius bucculatricis *(Ashmead). C. Rogadini: *Triraphis fusciceps *(Cresson). D. *Heterogamus longipendulatus *(van Achterberg) **comb. nov**. E. *Aleiodes *(*Chelonorhogas*) *convexus *van Achterberg. F. *Aleiodes *(*Aleiodes*) *albitibia *(Herrich-Schaeffer). G. *Spinaria armata *Ashmead. Yeliconini: H. *Yelicones fisheri *Areekul & Quicke.

Approximately one out of ten insect species is a parasitoid, that is, their larvae develop by feeding on or in other arthropods, which they eventually kill. Most parasitoid insects are hymenopterans, and an important fraction of these belong to the family Braconidae. Study of insect parasitoids is important in order to characterise their biodiversity, understand their evolution, and in some cases make use of their parasitic abilities for practical purposes, such as biological pest control [1]. In this paper we focus on the evolution of the Braconidae subfamily Rogadinae, a cosmopolitan and highly diverse group of Lepidoptera-parasitizing wasps with exclusively koinobiont biology, i.e., allowing the recovery and subsequent temporary development of the host after attack [2-4]. The rogadines are currently divided into four tribes, the Clinocentrini, Stiropiini, Yeliconini and Rogadini [5-7] (Figure 1). The latter is by far the most diverse as it contains the highly speciose and widely distributed genus *Aleiodes *Wesmael, with more than 300 of the approximately 500 species currently described for the subfamily [5-7]. Rogadines are currently defined only by a single biological synapomorphy, the 'mummification' of the host larvae [5], whereby the wasp deposits her egg (or eggs in a few cases) inside a host caterpillar which, after the parasitoid larva has completed feeding, turns into a variously hardened and tanned mummy within which the parasitoid pupates. It has been proposed that this complex strategy has resulted from selective pressures that are intimately related to the biology of a particular host species, and the results are apparent from the observed variability of mummy type within and between the tribes [8,9].

Despite the scant knowledge of host ranges within Rogadinae, a variety of lepidopteran host groups have been confirmed. Species of the Stiropiini, Clinocentrini and Yeliconini are only known to attack 'microlepidopteran' larvae [8,10,11], most of which are concealed feeders [12]. On the other hand, members of Rogadini, as currently recognised [13], attack both micro- and macrolepidopteran larvae [8], the latter generally having exposed feeding habits [12,14]. In *Aleiodes*, however, parasitism has been observed to occur almost exclusively on macrolepidopteran hosts, although some microlepidopterans with similar exposed feeding habits (e.g. species of Zygaenidae, Yponomeutidae and Pterophoridae) are also attacked by a few species, and only in rare cases does *Aleiodes *attack macrolepidopterans living in semi-concealed situations [7].

The few molecular phylogenetic analyses that have examined the evolutionary relationships within the Rogadinae have been constrained by limited taxon sampling. Chen et al. [15] presented the only molecular phylogenetic study devoted exclusively to the subfamily but included only 20 species collectively representing nine genera, and the work was based on a single DNA sequence fragment (449–482 bp of the D2 variable region of the 28S rDNA gene). Although most of the relationships investigated could not be resolved with confidence, the Rogadini and its subtribes were not recovered as monophyletic.

More recently, Zaldívar-Riverón et al. [16] carried out a simultaneous molecular and morphological phylogenetic analysis among the cyclostome subfamilies of Braconidae (braconids have traditionally been divided into two major groups, the cyclostomes and non-cyclostomes, based on the presence/absence of an oral opening formed by a ventrally concave clypeus). They employed two gene regions (28S rRNA and COI mtDNA genes) and included representatives of 15 rogadine genera and 19 species. They recovered a weakly supported monophyletic Rogadinae [16] and some relationships that were not concordant with the existing classification of the group, including a non-monophyletic Rogadini, with most members recovered as the sister group of Stiropiini, and with an *Aleiodes *clade sister to Yeliconini.

Neither of the above studies dealt with the evolution of mummification nor host ranges, nor did they estimate divergence times. Here we reconstruct the first extensive phylogeny for the Rogadinae using a combination of cytochrome oxidase I (COI) mtDNA and 28S rDNA gene sequences. Based on the trees obtained, we evaluate the current higher-level classification of the Rogadinae. We also estimate times of divergence and assess the evolution of host ranges and mummification within the group. Finally, we discuss the driving force for the extraordinary diversification within *Aleiodes*

## Results

### Molecular phylogenies

**Figure 2:**
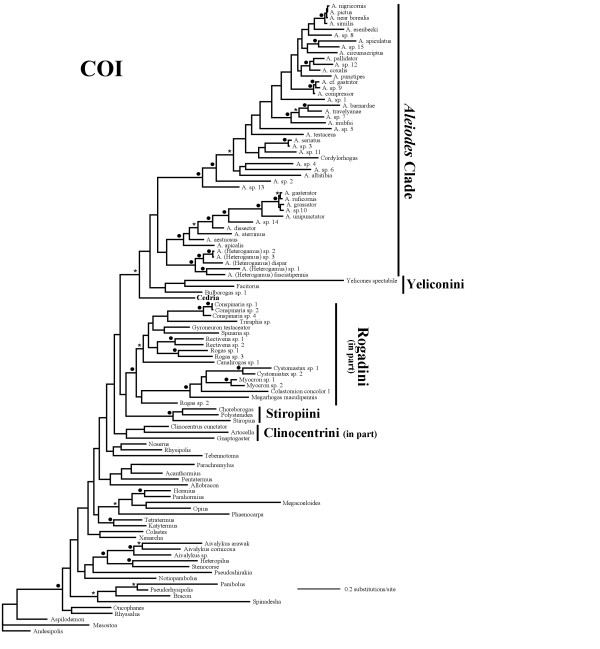
**Bayesian phylogram derived from the COI data set**. Bayesian phylogram obtained from the analysis of the COI data set (30 million generations; burn-in = 20 million generations). Parentheses and black circles above branches indicate clades supported by posterior probabilities from 0.8–0.94 and ≥ 0.95, respectively. *Cedria*, which was excluded from the simultaneous analysis (see results), appears in bold.

**Figure 3:**
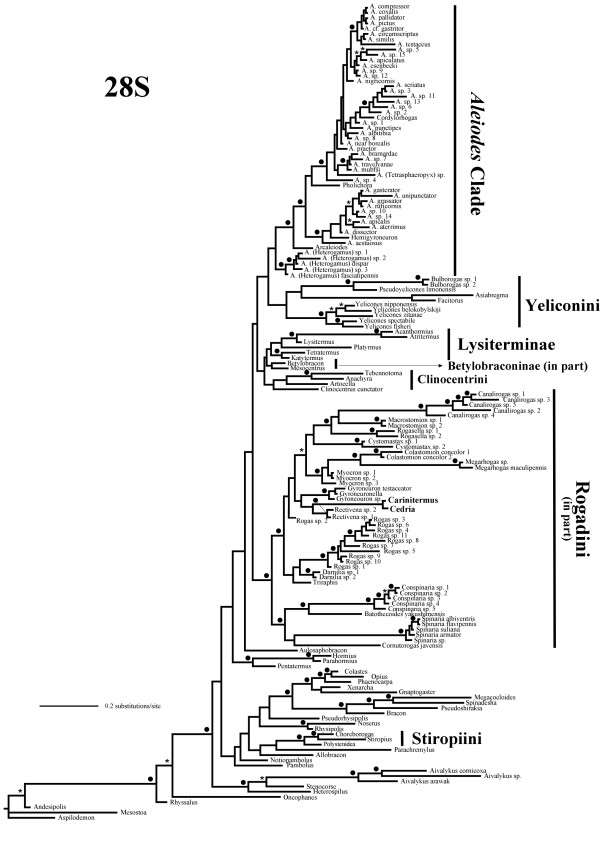
**Bayesian phylogram derived from the 28S data set**. Bayesian phylogram obtained from the analysis of the 28S data set (30 million generations; burn-in = 20 million generations). Parentheses and black circles above branches indicate clades supported by posterior probabilities from 0.8–0.94 and ≥ 0.95, respectively. *Cedria *and *Carinitermus*, which were excluded from the simultaneous analysis (see results), appear in bold.

The Bayesian topologies obtained from the separate 28S and COI analyses are shown in Figures 2 and 3. The 28S topology has a considerably higher number of clades with Bayesian posterior probabilities (PP) ≥ 0.8 compared to the COI one (28S: PP 0.8–0.94 = 72, PP ≥ 0.95 = 61; COI: PP ≥ 0.8–0.94 = 34, PP ≥ 0.95 = 44). The two separate analyses recover a number of similar relationships, and there are no significantly supported clades in conflict between them. Among the similar relationships recovered by both separate analyses are a clade with the species of *Aleiodes *Wesmael, *Cordylorhogas *Enderlein, *Hemigyroneuron *Baker and *Pholichora *Achterberg ('*Aleiodes *clade' hereinafter) (PP: 28S = 0.69; COI = 0.45), a Yeliconini + *Aleiodes *clade (PP: 28S = 0.7; COI = 0.52), and a clade with the remaining Rogadini genera (PP: 28S = 1.0; COI = 0.96).

The phylogenetic affinities of the two examined members of the non-rogadine tribe Cedriini, *Cedria *Wilkinson and *Carinitermus *Achterberg, vary considerably between the separate and simultaneous analyses. These taxa appear weakly supported in the simultaneous analysis at the base of the *Aleiodes *+ Yeliconini clade, a highly unlikely relationship considering the extreme difference in morphological and life history features. We therefore carried out an additional simultaneous analysis excluding both *Cedria *and *Carinitermus*. The topology of the latter analysis was similar to the one that included the above two genera, and thus we only show the topology that excluded them.

**Figure 4:**
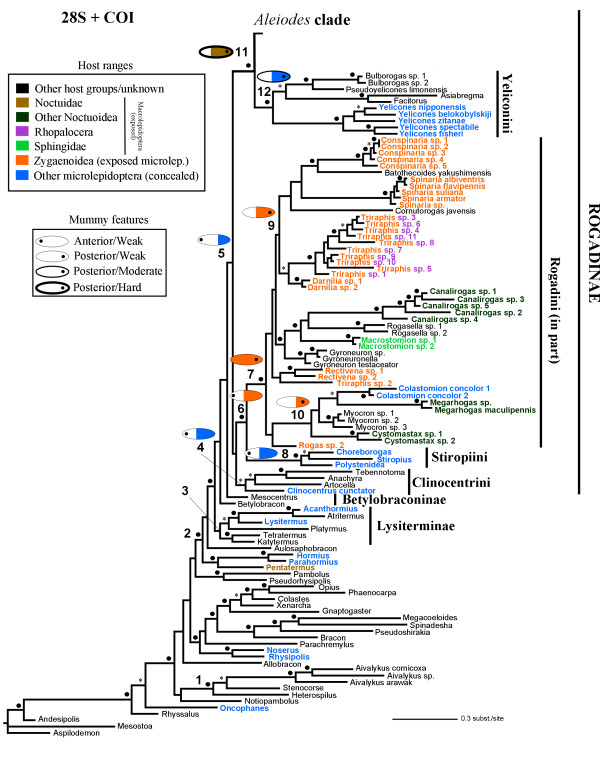
**Bayesian phylogram derived from the 28S + COI data sets**. Bayesian phylogram obtained from the simultaneous (28S + COI) analysis (30 million generations; burn-in = 20 million generations). Asterisks and black circles above branches indicate clades supported by posterior probabilities of 0.8–0.94 and ≥ 0.95, respectively. Host records for the terminal taxa included are indicated in colours. Numbered clades correspond to selected groups investigated for molecular dating and ancestral reconstruction analyses (see also Tables 1 and 2). The ancestral states of selected clades that were recovered by the Bayesian method are illustrated, with the coloured length representing the ancestral posterior probabilities (APP) obtained for the host ranges character (see APP values obtained for the three ancestral character reconstructions examined in Table 2).

**Figure 5:**
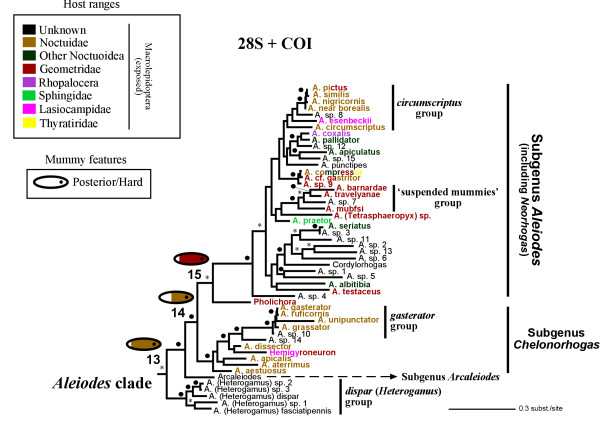
**Bayesian phylogram derived from the 28S + COI data sets**. Bayesian phylogram obtained from the simultaneous (28S + COI) analysis (30 million generations; burn-in = 20 million generations). Asterisks and black circles above branches indicate clades supported by posterior probabilities of 0.8–0.94 and ≥ 0.95, respectively. Host records for the terminal taxa included are indicated in colours. Numbered clades correspond to selected groups investigated for molecular dating and ancestral reconstruction analyses (see also Tables 1 and 2). The ancestral states of selected clades that were recovered by the Bayesian method are illustrated, with the coloured length representing the ancestral posterior probabilities (APP) obtained for the host ranges character (see APP values obtained for the three ancestral character reconstructions examined in Table 2).

Figures 4 and 5 show the 50% majority rule consensus tree derived from simultaneous analysis of the two gene fragments. This has considerably more clades with PP ≥ 0.8 (PP 0.8–0.94 = 101; PP ≥ 0.95 = 80) than either of the topologies from the separate analyses. Among the significantly supported relationships that are congruent with the current classification of Rogadinae are the monophylies of Clinocentrini, Stiropiini, and Yeliconini (PPs = 0.93, 1.0, and 1.0, respectively). The Rogadinae and Lysiterminae were also found to be monophyletic, though with weak support (PPs = 0.35 and 0.75, respectively). Some other relationships, however, are in disagreement with current taxonomy. Members of the Rogadini (*sensu *van Achterberg [13]) are grouped in two separate clades. One of these, with significant support (PP = 1.0), consists largely of the included species of *Aleiodes *(PP = 0.86), which form the sister group to the Yeliconini (PP = 1.0). The second clade (PP = 0.4) contains most of the remaining Rogadini species (PP = 1.0) as sister group to the Stiropiini (PP = 1.0) and with the Clinocentrini (PP = 0.94) at the base. The Betylobraconinae (*sensu *Belokobylskij et al. [17]) was not recovered as monophyletic, with the Australian *Mesocentrus *Szépligeti and *Betylobracon *Tobias instead forming a grade between Lysiterminae and Rogadinae, and with *Aulosaphobracon *as sister group of all of them (PP = 0.53).

The *Aleiodes *clade additionally includes members of another three genera, *Pholichora*, *Hemigyroneuron *and *Cordylorhogas*. Relatively few of the species groups proposed by Fortier and Shaw [18] for which we included more than one representative were significantly supported, but rather the relationships were congruent with the currently recognised subgeneric classification of *Aleiodes *(Figure 5). Their *A. dispar *(Haliday) group (PP = 1.0), with the exclusion of *A. punctipes *(Thomson), forms the sister group to a clade with the subgenus *Arcaleiodes *+ the remaining *Aleiodes *species (PP = 1.0). The two other groups proposed by Fortier and Shaw [18] that were recovered were the *A. gasterator *(Jurine) and the *A. circumscriptus *(Nees) [including *A. esenbeckii *(Hartig) and *A. *sp. 8] groups. The species known to produce suspended mummies [9] appeared significantly supported as monophyletic (PP = 1.0). The 28S motif TGCGT located at positions 264–268 [stems 3i'-3j of the 28S braconid secondary structure model of Gillespie et al. [19]] in our alignment is highly conserved within the Rogadinae and within the Braconidae in general, and is retained in the *dispar *group; however, the homologous positions in the remaining *Aleiodes *species as well as in *Pholichora*, *Hemigyroneuron *and *Cordylorhogas *display a derived AGCGT motif.

A clade with the members of the subtribe Spinariina with the inclusion of *Cornutorogas *(PP = 0.56) appears deeply nested within the clade including the remaining members of Rogadini (defined in a restricted sense, i.e. excluding the species assigned to *Aleiodes *together with *Pholichora*, *Hemigyroneuron *and *Cordylorhogas*). The Neotropical species of *Triraphis *constitute a significantly supported clade (PP = 1.0) that excludes the only Old World species of this genus included here.

None of the credible set of trees obtained from the simultaneous analysis recovered *Aleiodes *as currently constituted as monophyletic, nor a monophyletic *Triraphis*, Rogadini (including *Aleiodes*) and Rogadina (i.e. excluding Spinariina), and thus these alternative hypotheses are statistically rejected.

### Ages of diversification in Rogadinae

**Figure 6:**
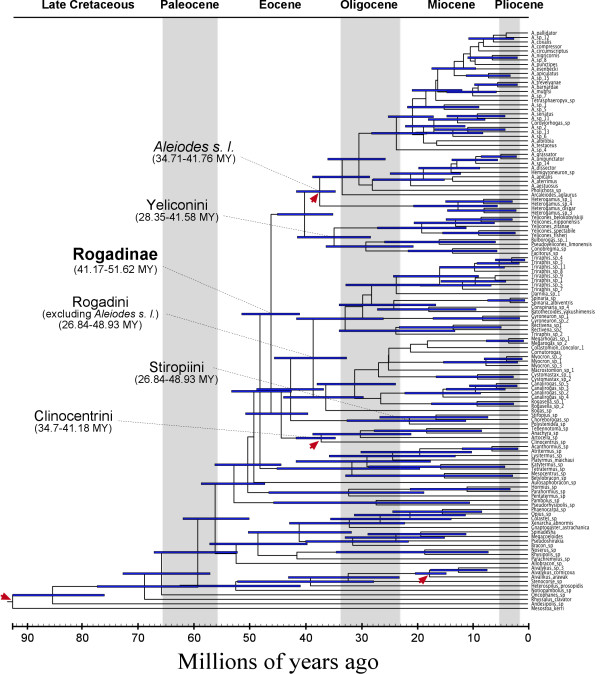
Times of divergence (including 95% confidence intervals for each estimate) derived from the Bayesian relaxed phylogenetic analysis using the program BEAST and including node calibration for the MRCAs of the Clinocentrini, *Aleiodes s. l*. *Aivalykus*, and for the root of the tree (see methods for details about the age calibrations employed). The nodes representing the higher taxonomic groups within the Rogadinae are indicated.

The times of divergence of selected clades (mean, standard deviation and range) using the penalised likelihood (PL) and Bayesian relaxed phylogenetics (RP) approaches are given in Table 1 and the chronogram for RP is shown in Figure 6.

**Table 1:** Estimates of divergence times for selected clades based on the penalised likelihood and relaxed phylogenetic analyses.

Most recent common ancestor	Penalised likelihood			Relaxed phylogenetics	
	Mean	SD	Range	Mean	Range

1. Doryctinae (South America)	40.17 38.12	3.87 3.71	30.72–49.5 29.11–49.96	39.21 38.6	27.78–52.38 25.74–49.86
2. Rogadinae + Betylobraconinae + Lysiterminae + Hormiinae	48.25 45.14	2.09 3.13	43.49–53.34 39.4–60.36	50.53 47.15	44.52–56.48 40.03–52.4
3. Lysiterminae	41.59 38.51	3.87 4.21	32.96–48.53 29.39–57.39	29.01 29.22	17.53–41.81 15.01–42.26
4. Clinocentrini	35.88 34.87	1.25 1.11	34.7–39.16 34.7–44.47	37.28 37.36	34.7–41.81 34.7–41.18
5. Rogadinae	41.59 38.11	1.15 1.88	37.48–44.4 36.1–51.55	46.34 44.61	41.17–51.62 38.86–49.38
6. Rogadini (excl. *Aleiodes s. l.*) + Stiropiini	36.76 33.89	2.31 1.84	29.8–43.73 22.71–39.29	42.79 -^a^	26.84–48.93 -^a^
7. Rogadini (excl. *Aleiodes s. l.*) ^b^	27.64 25.58	2.61 2.34	20.47–33.96 21.03–34.41	36.44 37.33	29.66–44.09 31.04–42.76
8. Stiropiini	15.33 14.34	2.7 1.93	10.28–30.45 9.55–20.41	21.36 19.15	11.16–32.62 9.17–31.02
9. 'Zygaenoid hosts' clade ^c^	23.77 22.01	2.95 2.87	14.24–32.01 14.45–33.44	28.17 30.33	-^d^ -^d^
10. *Colastomion *+ *Cystomastax *+ *Megarhogas *+ *Myocron *clade	25.27 23.37	2.6 2.35	18.5–32.39 16.58–30.52	26.92 25.94	-^d^ -^d^
11. *Aleiodes *clade + Yeliconini	37.04 28.34	1 2.88	35.11–40 22.76–40.22	40.29 38.79	35.15–45.18 31.93–44.17
12. Yeliconini	31.54 23.09	1.76 2.93	26.74–35.35 16.79–36.12	34.95 33.44	28.35–41.58 25.14–40.06
13. '*Aleiodes*' clade	34.72 23.89	0.23 2.7	34.7–37.04 17.98–38.07	37.54 36.12	34.71–41.76 28.12–40.74
14. Subgenera *Aleiodes *+ *Chelonorhogas *^e^	20.7 13.26	3.09 2.65	14.33–28.47 7.82–25.74	30.46 29.42	25.69–36.09 23.02–35.12
15. Subgenus *Aleiodes*	15.23 9.77	3.64 3.02	7.9–25.16 5.23–25.52	23.71 22.33	18.25–28.18 15.73–28.61

The penalised likelihood (PL) analyses used a sample of 100 postburn-in trees derived from the two 28S + COI Bayesian searches. The relaxed phylogenetic (RP) analyses were based on eight independent runs of 100 million generations each. Numbers on the first column correspond to selected clades indicated in the 28S + COI Bayesian topology (see Figures 4 and 5). Values on the first and second lines for each of the selected clades correspond to divergence times parameters obtained including and excluding calibration of *Aleiodes s. l.*, respectively.

^a ^Relationship not recovered.

^b ^Relationship present in 96 out of the 100 sampled trees selected for the PL analyses.

^c ^Relationship present in 99 out of the 100 sampled trees selected for the PL analyses.

^d ^Range absent in the RP analyses.

^e ^Also including *Cordylorhogas*, *Hemigyroneuron*, and *Pholichora*.

^f ^Also including *Cordylorhogas.*

Because of the uncertainty associated with the calibration of the *Aleiodes s. l. *clade (see Figure 6 for identifying this clade), we ran PL both including (PLi*A*) and excluding (PLe*A*) the calibration associated to *Aleiodes s. l. *Ten of the fifteen clades that were examined in the two PL analyses have a PP value equal to or higher than 0.95 in our 28S + COI Bayesian phylogeny, and thirteen of them higher than 0.8 (Figures 4 and 5). The taxonomic composition of the clades examined using the PL approach was the same as for the 100 sampled trees except in two relationships, which were not present in four and one of the sampled trees, respectively (see Table 1).

The RP analyses including (RPi*A*) and excluding (RPe*A*) the *Aleiodes s. l. *node calibration both recovered most of the relationships found using MrBayes except for the Rogadini + Stiropiini clade, which was not recovered in the RPe*A *analysis. The age estimates of clades obtained by the two RP analyses are generally similar; thus, we only show the ultrametric tree derived from the RPi*A *analysis, because it incorporated more node calibrations (Figure 6).

The age estimates derived from the two RP analyses are generally earlier than those from the PL ones (Table 1). The lineage that led to the extant members of the Rogadinae is estimated to have diverged during the late to mid Eocene (36.1 to 51.62 MYA). Among the rogadine tribes, the age of the most recent common ancestor (MRCA) of the Stiropiini and Yeliconini diversified between the late to mid Miocene (9.55 to 32.62 MYA) and the late Eocene to early Miocene (16.79 to 41.58 MYA), respectively. The MRCA of the Rogadini (excluding the *Aleiodes *clade) on the other hand was estimated to diverge during the late Miocene to mid Eocene (21.03 to 44.09 MYA). Of the four molecular dating analyses performed, the PLe*A *had a considerably younger age estimate for the origin of the MRCA of the *Aleiodes *clade (17.98 to 38.07 MYA; late Miocene to early Eocene) in comparison to the remaining PL and RP analyses (28.12 to 41.76 MYA; mid Oligocene to mid Eocene).

### Evolution of host ranges and mummification

The MP and Bayesian ancestral states of selected clades for the three biological features examined – host and site of emergence and hardening of mummy – are shown in Table 2. The Rogadinae ancestral condition for the site of emergence from the mummified host was recovered as equivocal with MP but anterior with the Bayesian method, though its ancestral posterior probability (APP) was very low (APP = 0.21). The Stiropiini and the Rogadini + Stiropini clades had an equivocal and anterior emergence as ancestral conditions with MP and Bayesian methods (APPs = 0.83 and 0.44, respectively). The ancestral reconstructions of the basal Rogadinae clades for the degree of hardness of the mummy were recovered as equivocal (MP) and with low APP values. Forming a hard mummy, however, appears marginally as nonsignificant in the *Aleiodes *clade. The latter condition also appears to have evolved independently in a few members of the Rogadini clade (*Cystomastax *and some species of *Triraphis*) and in some species of *Clinocentrus*.

**Table 2:** MP and Bayesian ancestral reconstructions of the host ranges and mummy features.

Clade	Host ranges		Site of emergence from mummy		Hardening of mummy	
	MP	Bayesian	MP	Bayesian	MP	Bayesian

4. Clinocentrini	Microlep.	Microlep. (0.6)	Equivocal	Anterior (0.8)	Equivocal	Moderate (0.42)
5. Rogadinae	Microlep.	Microlep. (0.3)	Equivocal	Anterior (0.21)	Equivocal	Weak (0.22)
6. Rogadini (excl. *Aleiodes *clade) + Stiropiini	Microlep.	Zygaenoidea (0.59)	Equivocal	Anterior (0.44)	Equivocal	Weak (0.71)
7. Rogadini (excl. *Aleiodes *clade)	Zygaenoidea	Zygaenoidea (0.98)	Equivocal	Posterior (0.82)	Equivocal	Weak (0.66)
8. Stiropiini	Microlep.	Microlep. (0.52)	Equivocal	Anterior (0.83)	Equivocal	Weak (0.9)
9. 'Zygaenoid host' clade	Zygaenoidea	Zygaenoidea (0.65)	Posterior	Posterior (0.34)	Moderate	Weak (0.44)
10. '*Colastomion' *clade	Zygaenoidea	Zygaenoidea (0.43)	Equivocal	Posterior (0.4)	Weak	Weak (0.64)
11. *Aleiodes *+ Yeliconini	Microlep.	Noctuidae (0.7)	Equivocal	Posterior (0.86)	Equivocal	Moderate (0.41)
12. Yeliconini	Microlep.	Microlep. (0.52)	Equivocal	Posterior (0.47)	Equivocal	Moderate (0.81)
13. *Aleiodes *clade	Equivocal	Noctuidae (0.88)	Equivocal	Posterior (0.74)	Equivocal	Hard (0.9)
14. *Aleiodes *clade excl. *Arcaleiodes *and *A. dispar *group	Equivocal	Noctuidae (0.62)	Posterior	Posterior (0.95)	Hard	Hard (1.0)
15. *Aleiodes *clade excl. *A. dispar *group, *Arcaleiodes *and *Chelonorhogas*	Geometridae	Geometridae (0.84)	Posterior	Posterior (0.85)	Hard	Hard (0.95)

Maximum parsimony and Bayesian (using the BayesTraits program) ancestral reconstructions of the three biological features examined in this study. Ancestral posterior probabilities were obtained by multiplying the mean ancestral character state probability of the selected node across all trees by the portion of the trees that recovered the node involved. Numbers on the first column correspond to selected clades that are indicated in the 28S + COI Bayesian topology (see Figures 4 and 5).

Use of microlepidopteran hosts was recovered by both methods as the ancestral condition for the Rogadinae, but only with a low APP with the Bayesian method. In any case, a shift to attack macrolepidopteran hosts appears to have occurred independently in various lineages within the Rogadini clade and once more in the *Aleiodes *clade. Moreover, our analyses show an expansion to attack various lepidopteran host groups in the *Aleiodes *clade, with the basal groups generally parasitising Noctuidae species whereas the derived ones attack other macrolepidopteran groups, including other families of Noctuoidea (e.g. Notodontidae, Lymantriidae, Arctiidae) as well as species of Geometridae, Thyratiridae, Lasiocampidae, Satyridae, Hesperiidae and Sphingidae, and rarely also a few microlepidopterans with exposed larval feeding habits (see also [7]).

## Discussion and conclusion

### Phylogenetic relationships and taxonomic inferences

Here we present the most extensive phylogenetic analysis within the Rogadinae to date based on DNA sequence data. Our study covered representatives of the different supraspecific taxa within the subfamily, as well as a number of putatively closely related groups that have previously been considered to belong to this group (viz. Betylobraconinae, Lysiterminae, *Pentatermus *Hedqvist; e.g. [13]). Our simultaneous analysis recovered a monophyletic Rogadinae as currently recognised, though with a non-significant PP. This, and the placement of the betylobraconines *Mesocentrus *and *Betylobracon *at the base of the Rogadinae clade leave the actual extent of the subfamily unresolved and probably the Betylobraconinae ought to be synonymised with the Rogadinae, especially if it were found that they mummify their hosts. Further work is also needed to confirm the phylogenetic position of the Clinocentrini. Unlike previous studies that recovered Clinocentrini at the base of the Rogadinae [15,16], in our study this tribe appeared weakly supported as sister group of the Stiropiini + Rogadini clade.

In the separate 28S and COI analyses as well as in the simultaneous analysis, the *Aleiodes *clade appears as the sister group to the Yeliconini, whereas in the latter two analyses a clade comprising the remaining genera of Rogadini (i.e. in the new sense of excluding *Aleiodes *and its close relatives) together with the Stiropiini was also recovered (Figures 2, 3, 4, 5, respectively). Since these relationships are consistent between the two gene markers, we propose the new tribe Aleiodini **nov. **for the members of the *Aleiodes *clade, within which we recognise two genera, *Aleiodes *and *Heterogamus ***stat. rev. **(see below).

The relationships recovered within *Aleiodes s. l. *were concordant with its subgeneric classification [13] except for the only examined member of the subgenus *Neorhogas*, which appeared deeply nested within a clade with the members of the subgenus *Aleiodes*. Of the species groups proposed for *Aleiodes *by Fortier and Shaw [18] based on morphological evidence alone, only the *A. dispar*, *A. gasterator *and *A. circumscriptus *groups were partially congruent with our molecular phylogeny. Of these groups, the *A. dispar *group should be treated as a separate genus according to its phylogenetic position and morphological distinctiveness [*Heterogamus ***stat. rev.**: type species *A. *(*H.*) *crypticornis *Wesmael = *H. dispar *(Haliday)]. Moreover, the derived 28S AGCGT motif present in *Aleiodes s.s. *is absent in *Heterogamus*. Members of *Heterogamus *can be morphologically distinguished from *Aleiodes s. s. *by having a considerably elongated hind trochantellus. Also, *Heterogamus *species all have forewing vein r relatively long in relation to vein 3-SR, though this state is also displayed by a range of *Aleiodes s. s. *species. An elongate head with a low clypeus (below lower level of eyes), narrow and usually (in females) somewhat banded wings, and long pronotum are also distinctive features in *Heterogamus*, though these are also occasionally present in some species of *Aleiodes *(Shaw and van Achterberg, unpubl. data). Further, the venom gland apparatus in examined members of *Heterogamus *appears to be less modified than those present in the species of *Aleiodes s. s. *[20], which is congruent with the basal position recovered for this taxon within Aleiodini.

The three previously-recognised genera that were recovered as nested within *Aleiodes s. s.*, i.e. *Pholichora*, *Hemigyroneuron *and *Cordylorhogas*, are distinguished by the possession of various derived morphological features (e.g. vein cu-a of fore wing strongly curved in *Hemigyroneuron*, vein 1-1A curved subapically and subbasal cell with a pair of patches in *Pholichora*, and posterior corner of first metasomal tergite acutely protruding and second tergite with crest-like lateral carina in *Cordylorhogas *[13]). However, these genera also possess morphologies characteristic of *Aleiodes*, such as tarsal claws without a basal lobe, setose hind tibial spurs, and otherwise similar wing venation and metasomal carination [13]. Also *Pholichora*, *Hemigyroneuron *and *Cordylorhogas *have the derived AGCGT 28S rDNA motiff (see above). Based on the latter information and our molecular phylogeny, we therefore synonymise the above three genera (*Hemigyroneuron*: type species *H. speciosus *Baker; *Pholichora*: type species *Hemigyroneuron madagascariensis *Granger; *Cordylorhogas*: type species *C. trifasciatus *Enderlein) with *Aleiodes *(**syn. nov.**), and all species currently classified within each of these are hereby transferred to *Aleiodes *(**comb. nov.**). We therefore propose that *Aleiodes s. s. *should be represented by the subgenera *Aleiodes *(including *Neorhogas *and *Cordylorhogas*), *Chelonorhogas *(including *Hemigyroneuron*), *Pholichora *and *Arcaleiodes*.

Two further relationships recovered here are also supported by features of the venom gland apparatus [20]. One of them, supported by the presence of a hard secondary venom duct with filaments, consistently shows that Spinariina is actually a derived group within the Rogadini, and thus the subtribal names employed within Rogadini (viz. Rogadina and Spinariina) should be eliminated. The other relationship, supported by the presence of distinct secondary venom duct branching patterns, suggests a polyphyletic *Triraphis*, with the American species being more closely related to other members of the Rogadini rather than to the only examined Old World species of this genus. Members of *Triraphis *from the New World had previously been assigned to *Rogas *(e.g. [20,21]); however, a recent study based on van Achterberg's [13] definition of rogadine genera revealed that all the examined species from the New World previously assigned to *Rogas *actually belong to *Triraphis *(*sensu lato*) and that *Rogas *may be absent from this region [22].

### Ages of diversification in the Rogadinae

The oldest cyclostome fossil that has been found to date (93 MYA [23]) has a considerably more recent age than the times of origin estimated for Ditrysia, which is the lepidopteran group that comprises all the taxa attacked by rogadines and that has been calculated to have originated 157.6 to 191.9 MYA [24]. The available fossil evidence thus indicates that the Rogadinae originated considerably later than its host group, with our molecular dating estimates showing that its MRCA originated during the mid to late Eocene. Our molecular dating estimates also show that the Aleiodini has a more recent origin (late Miocene to mid Eocene) than the origins estimated for two of its most frequently attacked lepidopteran families, Geometridae (48–62 MYA; mid Eocene to mid Paleocene [25]) and Noctuidae (60.7 to 113.4 MYA; mid Paleocene to mid Cretaceous [24]). Species of the remaining rogadine tribes often attack members of various basal ditrysian families (i.e. microlepidopterans), and thus their origins also are considerably more recent than those estimated for their hosts.

The short internal branches and low support for several of the relationships within the subgenus *Aleiodes*, which comprises most of the known diversity within the genus suggests a rapid radiation, although other factors, such as lower phylogenetic signal may also explain this pattern [26,27]. The inclusion of additional markers should help to clarify whether there has been a rapid radiation within the group. A rapid radiation has also been proposed within Braconidae for the microgastrine group of genera (40–50 MYA; [28]).

### Evolution of mummification and emergence characteristics

The lack of host records and/or preserved host mummies for several rogadine genera, as well as our failure to recover some key relationships (e.g. placement of Clinocentrini), prevent us from drawing strong conclusions about the evolution of mummification characteristics within the subfamily. However, whether Clinocentrini is the sister group to the Rogadini + Stiropiini clade or the sister group of the remaining rogadine tribes, a weak hardening of the mummy is most likely to be the ancestral condition for the subfamily. Producing a hard mummy could have evolved repeatedly in separate lineages, though this condition is mainly found in species of the Aleiodini. An anterior site of emergence from the mummified host would be the most plausible ancestral state if the Clinocentrini is confirmed as the sister group of the remaining rogadine tribes.

The dorsal emergence from the mummified host observed in all examined species of Stiropiini, Yeliconini and Aleiodini presumably occurs in response to the avoidance of physical obstruction at other orientations. In the Stiropiini this may occur because the host is prepupal in a cocoon ventrally attached to a hard substrate, and in Yeliconini because the prepupal mummy itself is curved under at both extremities [8]. The particular shift to exactly posterodorsal emergence in members of Aleiodini might be associated with the anteroventral fixing of the mummy (typically formed from an only partly grown caterpillar) to a hard substrate, which creates a barrier to emergence through the mummy's more contracted and harder anterior end.

This presumably derived feature is retained in the Aleiodini even in species that (apparently secondarily) have adopted a mummification procedure that avoids this source of obstruction (e.g. *A. pallidator*, which produces a loose mummy in a host-produced roomy silk chamber; and species of the 'suspended mummy' clade within *Aleiodes s. s. *[9]). Posterodorsal emergence orientations are also retained in the few gregarious species of *Aleiodes *[8], in marked contrast with the more chaotic emergence seen in gregarious genera of Rogadini *s. s. *such as *Colastomion *and *Macrostomion *[8,29]. On the other hand, in the species of the Clinocentrini and most Rogadini *s. s. *the host is killed in its cocoon (albeit sometimes precocious in *Clinocentrus *[2]), with typically no radial impediment, and consequently the emergence position shows no dorso-ventral restriction, but rather appears to occur more or less at random. However, in *Rogas*, in which the configuration of the prepupal mummy positions the host head capsule ventrally, and in *Triraphis*, in which the part grown larval mummy is attached ventrally to a substrate, dorsal emergence is seen.

The degree of hardening of the mummy produced by rogadine species from their host's integument appears to owe much to the need for protection of the parasitoid metamorphosing within it bearing in mind that there will be a cost in providing it. The known hosts of the Stiropiini, Clinocentrini, Yeliconini and most members of the Rogadini are killed as prepupae, i.e. in prepared pupation sites, which probably offers substantial protection to the parasitoid larvae and, especially when emergence is rapid, little selection for the mummy to be especially hard. Thus, relatively frail and pale-coloured mummies are normal. A revealing exception is seen in the European species *Triraphis tricolor *(Wesmael), whose part grown host mummies are either fixed naked to a tree leaf and emerge rapidly, when they are pale greenish yellow and relatively frail, or fall from the leaf to overwinter on the ground, in which case they are very well-tanned, dark brown and tough (MRS, unpublished data).

Most known hosts of the Aleiodini, on the other hand, are killed during early larval instars, and lack the protection of the host's pupation site, with the result that the mummy itself plays a more important role. Thus, the mummies of these species are usually toughened and often strongly darkened, whether or not the parasitoid diapauses in them.

### Evolution of host ranges

Use of microlepidopteran hosts was generally recovered by the MP and Bayesian methods as the ancestral condition for both the Rogadinae and the Rogadini + Stiropiini clade. Thus, our results suggest that a shift from attacking semi-concealed microlepidopteran to exposed microlepidopteran (Zygaenoidea) or macrolepidopteran host taxa has independently evolved in various lineages within the Rogadinae.

Our ancestral reconstruction of host ranges and divergence time estimates contradict Fortier and Shaw's [18] hypothesis of coevolution between *Aleiodes s. l. *and its lepidopteran hosts. Based on their morphological estimate of phylogeny, they suggested that putatively basal macrolepidopteran families (non-catocaline Noctuidae, Sphingidae, Notodontinae) are attacked by basal species of *Aleiodes*, whilst the most derived hosts ('trifine' Noctuidae) are attacked by derived parasitoid taxa. In our molecular phylogenetic estimate, however, Fortier and Shaw's [18] supposedly derived *A. dispar *and *A. gasterator *species groups appear instead, with significant support, to be at the base of Aleiodini, and most of the species belonging to their proposed basal *A. circumscriptus*, *A. pallidator*, *A. gastritor*, and *A. compressor *groups are recovered in derived clades.

Our results are more congruent with Shaw's [4,7] host ecology hypothesis that explains the apparently rapid radiation in *Aleiodes s. s*. In this hypothesis, the species of *Aleiodes s. l. *that broaden their host range by recruitment of new hosts, sometimes from additional lepidopteran families, promote subsequent radiation within the group. In contrast, other species of *Aleiodes *remain tied to phylogenetically restricted host taxa and so have less tendency to radiate. In our molecular phylogeny, the most basal clades (*Heterogamus*, the species assigned to the subgenus *Arcaleiodes *and the *A. gasterator *and other species groups of Fortier and Shaw [18] corresponding to the subgenus *Chelonorhogas sensu *van Achterberg [13]), indeed do not appear to be particularly species rich and seem to be mostly restricted to attacking only Noctuidae, though the hosts of *Heterogamus *and the subgenus *Arcaleiodes *remain unknown (old published records, cf. [30] repeated in Fortier and Shaw [18], are almost certainly erroneous and have never been repeated). In contrast, the intermediate and derived clades (subgenus *Aleiodes sensu *van Achterberg [13]) comprise most of the species diversity within the genus and exhibit a wide host range overall [7]. Within this, specialist use of microlepidoptera taxa such as *Zygaena *(Zygaenidae), *Ypsolopha *(Ypsolophidae) and some Pterophoridae, occurs infrequently, the species using these hosts apparently being very closely related to species that parasitise macrolepidoptera including morphologically similar species in similar feeding niches (MRS, unpubl. data). Thus, the use of microlepidoptera by *Aleiodes *appears to be secondary. Moreover, in temperate western European species (the biologically best known), the expansions of host range that have been hypothesised [4,7] to lead to speciation seem often to occur in species having more than one generation a year, which often use different and sometimes not closely related hosts successively. This plurivoltinism with 'discontinuous' host ranges as defined by Shaw [7] is seen much more frequently in species of the subgenus *Aleiodes *than in the subgenus *Chelonorhogas*. Members of the latter are mostly univoltine and have 'continuous' host ranges.

## Methods

### Taxon sampling

Our taxon sampling consisted of sequences from 34 genera and 118 species belonging to the previously recognised rogadine tribes Clinocentrini (4 genera, 4 spp.), Stiropiini (3 genera, 3 spp.), Yeliconini (*sensu *Belokobylskij et al. [17]; 5 genera, 10 spp.), and Rogadini (22 genera, 101 spp.). Within the Rogadini, the species-rich genus *Aleiodes *was represented by a total of 48 species. Our selection of the *Aleiodes *species included members from 13 of the 17 species-groups proposed by Fortier and Shaw [18] based on a morphological phylogenetic analysis that comprised 208 species. The species groups not represented in our study were the *A. gressitti *(Muesebeck), *A. procerus *Wesmael, *A. pulchripes *Wesmael and *A. ufei *Walley groups. Our species sampling also represents van Achterberg's [13] three proposed subgenera, *Aleiodes *Wesmael, *Chelonorhogas *Enderlein and *Neorhogas *Szépligeti. We also included a specimen assigned to the subgenus *Arcaleiodes *Chen & He (*sensu *Belokobyslkij [31]), which was originally described as a separate genus [15]. Moreover, we included a species belonging to the more recently erected *pilosus *species-group [= *Tetrasphaeropyx *Ashmead [32]] and 6 described and 15 undescribed morphologically diverse *Aleiodes *species without species-group assignation. Among these are four Afrotropical species (*A. mubfsi *Quicke & Shaw, *A. barnardae *Quicke & Shaw, *A. travelyanae *Quicke & Shaw, and *A. *sp. 7) characterised by leaving their mummified hosts suspended (known in three cases; unknown for *A. *sp. 7), which appear to form a separate species group probably related to the Asian *A. buzurae *He & Chen [9].

Monophyly of the Rogadinae was tested by adding 40 terminal taxa belonging to most of the currently recognised cyclostome braconid subfamilies. In particular, our outgroup sampling emphasised the small subfamilies Betylobraconinae, Lysiterminae, and Hormiinae, which appear to be closely related to the Rogadinae according to previous molecular [16] and morphological [5,33] phylogenetic analyses. All the recovered trees were rooted using *Mesostoa kerri *Austin & Wharton, of Mesostoinae. A Mesostoinae + Aphidiinae clade has been consistently recovered in previous studies as the sister group of the remaining cyclostomes [16,34]. The Aphidiinae was not represented in our study because the species of this group display marked sequence length variation in the sequenced 28S gene fragment, which considerably affects the length of the unambiguously aligned positions in the matrix.

The taxa examined, their localities and voucher and EMBL/GenBank accession numbers are listed in Additional file 1.

### Molecular data

The gene markers analysed comprised a ~650 bp fragment of the second and third domain regions of the nuclear 28S rRNA gene, and a 602 bp fragment of the cytochrome oxidase I (COI) mitochondrial DNA gene. Most of the sequences analysed were newly generated for this study, but some were previously published elsewhere by us (see Additional file 1). We also retrieved from GenBank the 28S sequence of *A. *(*Arcaleiodes*)*aglaurus *[15]. Genomic DNA was extracted from alcohol-preserved specimens and from dry-mounted material up to 15 years old. Detailed information on the DNA extraction and PCR protocols employed, primers selected and sequencing procedure of PCR products is given in Zaldívar-Riverón et al. [16].

### Sequence alignment

Both 28S and COI sequences were aligned by eye. COI alignment was confirmed by reference to the translated amino acid sequence. The sequence length variable 28S fragments were aligned following the braconid secondary structure model of Gillespie et al. [19]. The ambiguously aligned regions detected were further identified and characterised according to the categories proposed by Gillespie [35]. Unfortunately, the vast number of terminal taxa together with the extensive variation observed within most of the detected 28S unalignable regions precluded us from implementing the approach for recoding such regions proposed by Zaldívar-Riverón et al. [16]. Thus, the 11 unalignable regions that were delimited in our 28S alignment were excluded from the subsequent analyses. The 28S + COI matrix showing the braconid secondary structure model followed for the 28S sequences is given in Additional file 2. The above matrix and its resulting Bayesian topology can also be retrieved from the TreeBase web page (http://www.treebase.org; study accession number S2223).

### Phylogenetic analyses

Separate and simultaneous analyses were carried out for the 28S and COI data sets using the mixed-model Bayesian MCMC method as implemented in MrBayes version 3.1.2 [36]. Congruence between the two data sets was assessed by detecting the significantly supported clades [posterior probability (PP) ≥ 0.95] that were in conflict between the two separate analyses [37,38].

All the Bayesian analyses were run for 30 million generations in Bioportal (University of Oslo; https://www.bioportal.uio.no/). Each analysis consisted of two searches, sampling trees every 1000 generations and using four chains and default priors. The model of evolution selected for the two data sets was the GTR+I+Γ, which was determined based on the Akaike criterion implemented in MrModeltest version 2.0 [39]. Five different partitions were considered for the analyses, two for the stem and loop 28S regions, respectively, and the three remaining for the COI codon positions. Burn-in is usually determined by stationarity of the likelihoods of the trees sampled, which is usually reached quickly. This practice, however, may not work so well for large data sets, where the appropriate sampling of the posterior distribution of the tree topologies might take considerably longer [40]. We therefore took advantage of our large analyses and determined the duration of the relevant burn-in phases based on the constancy of the PPs for the 20 more unstable clades using the online program AWTY [41]. Based on this conservative criterion, the burn-in phase in all analyses was determined to be after 20 million generations. We then compared the topologies and PPs obtained in the two independent searches run for the separate and combined analyses. The independent searches were congruent in their topologies and PP clade values in the three analyses, and thus majority rule consensus trees (including compatible groups) of their pooled post burn-in trees were estimated. We considered clades as significantly supported if they had a PP ≥ 0.95 [42].

A Bayesian approach for hypothesis testing [38,43,44] was carried out to test the following hypotheses that were absent in the majority rule consensus tree derived from the simultaneous analysis of the two gene fragments: 1) monophyletic *Triraphis *Nees (*sensu *Valerio [22]), 2) monophyletic *Aleiodes *(including the species of the subgenera *Chelonorhogas*, *Neorhogas*, *Aleiodes *and *Arcaleiodes *and the species previously considered as *Heterogamus*, but excluding *Pholichora *and *Heterogamus*), 3) monophyletic Rogadini (*sensu *van Achterberg [13]), and 4) monophyletic Rogadina (considering *Cornutorogas *Chen, Belokobylskij, Achterberg & Whitfield and *Batothecoides *Watanabe as members of Spinariina, see results). A detailed explanation of how this Bayesian approach was implemented can be found in Zaldívar-Riverón et al. [45].

### Divergence dates of clades

The molecular dating of selected clades was estimated using two different relaxed molecular clock approaches: the semiparametric penalised likelihood (PL; Sanderson, 2002) and the Bayesian relaxed phylogenetics (RP [46]) methods.

The PL method was performed with r8s version 1.7 [47]. The range, mean and standard deviation for PL were obtained taking the last 50 trees (with their branch lengths) sampled from each of the two independent MrBayes runs (with 28S and COI combined; see above). The truncated Newton algorithm was used, and a cross validation [48] was first run on the majority rule consensus tree from the simultaneous Bayesian analysis to determine the optimal value of smoothing, which turned out to be 10. *Aspilodemon *Fischer was deleted from the trees examined to give a basal node separating *Mesostoa *Austin & Wharton from the clade with the remaining terminal taxa. The following terminal taxa were pruned from the trees in order to avoid near zero branch lengths: *A. *sp. near *borealis *(Thomson), *A. gasterator *(Jurine), *A. *cf. *gastritor *(Thunberg), *A. pictus *(Herrich-Schäffer), *A. praetor *(Reinhard), *A. ruficornis *(Herrich-Schäffer), *A. similis *(Curtis), *A. *(*Heterogamus*) sp. 2, *A. *spp 3, 9, and 10, *Bulborogas *sp. 1, *Canalirogas *sp. 1, *Colastomion concolor *(Szépligeti) specimen 2, species of *Conspinaria *Schulz except *C. *sp. 4, *Darnilia *sp. 2, *Gyroneuronella *sp., *Macrostomion *sp. 2, *Rogas *spp. 6 and 10, and species of *Spinaria *Brullé except *S. albiventris *Cameron and *S. *sp.

The RP method was performed using BEAST [49]. The data set was partitioned in four sets: 28S, and the first, second and third codon positions of COI. The same model of nucleotide substitution was used for each of these partitions: GTR+I+Γ (selected by MrModeltest; see above). Base frequencies were estimated by BEAST, and the number of categories for the gamma distribution was four. We included the same set of 129 taxa that we used for the PL method. The relaxed molecular clock model chosen was uncorrelated lognormal. The starting tree was obtained with PAUP* using Maximum Parsimony [50] and later linearised with r8s [47] with a tree height of 93 MYA (see below). We ran each analysis for 100 million generations in eight separate runs, and with a sampling frequency of 1/10,000 generations. Given the size of the resultant files, and to make sure that the sampled chains were stable, we discarded the first 80 million generations of each run (burn-in), which was more than enough according to what the chain inspector software Tracer v1.4 [51] suggested. We include the Beast files as Additional files 3 and 4.

### Fossil ages and node calibration

The correct identification of fossil taxa and an adequate selection of calibration nodes are two of the most critical steps in molecular dating estimation [52]. We fixed the most basal node and set two or three different calibration points based on the fossil taxa discussed below, which were selected after a detailed examination of a number of descriptions of cyclostome fossils. Several other cyclostome fossils have been described from different geological periods [53], but unfortunately the generic assignations for many of them are either incorrect or questionable.

The most basal node representing the cyclostome group was fixed in the PL and RP analyses using the age of *Protorhyssalus goldmani *Basibuyuk, Rasnitsyn, Achterberg, Fitton & Quicke [93 million years ago (MYA) [23]]. An apparently closely related fossil, *Protorhyssalodes arnaudi *Perrichot, Nel & Quicke, of very similar age has been recently confirmed to be a cyclostome and is the oldest confirmed member of this group that has been found to date (Perrichot, Nel and Quicke, submitted).

The MRCA of *Aivalykus *Nixon was calibrated based on the fossil of *A. dominicanus *Zuparko & Poinar [54], which has an age of 15–20 MYA according to the estimated age of the Dominican amber [55]. Three species of *Aivalykus *were included in the analyses in order to have a better representation of the oldest lineage within this genus. The MRCA of *Aivalykus *was calibrated with a minimum age of 15 MYA in the PL analyses, whereas in the RP analyses this was constrained to a normal prior distribution with a lower bound of 15 MYA (5%) and a higher bound of 20 MYA (95%). We chose a normal distribution to reflect the similar probability of the two bounds, and also to account for the uncertainty associated with the long branch sustaining the node of *Aivalykus*' MRCA. That is, the dated fossil might be from a time point considerably older than *Aivalykus*' MRCA because of the long branch previous to this MRCA (Figure 4), and therefore *Aivalykus*' MRCA could not only be older than the fossil, as is always the case due to the paucity of the fossil record, but also younger.

We also selected the fossil ages of three rogadine species that were described from the Baltic amber [56], *Clinocentrus latitator *Brues, *C. latipennis *Brues, and *Rhogas *(= *Rogas*)*fritschii *Brues. Unfortunately, these fossils were destroyed during the Second World War and thus we could not confirm the correct assignation of these taxa. A detailed inspection of the original descriptions and the Figures presented therein suggest the correct assignation of the two first species within *Clinocentrus*, though they show some atypical features that are absent in the extant species of the genus. In the case of the fossil specimen assigned to *Rogas*, its description unambiguously places it within the Rogadinae but does not help to confirm its generic assignation because is not clear whether it has the only known external morphological feature known to distinguish *Rogas *from *Aleiodes sensu *van Achterberg [13], i.e. tarsal claws with a basal lobe present. However, an examination of Brues' descriptions of other species assigned by him to *Rhogas *revealed that his concept of the genus was an idea (prevalent at the time) that encompassed *Aleiodes sensu *van Achterberg [13]. For instance, *R. bakeri *Brues and *R*. *ecuadoriensis *Brues have been subsequently reclassified as *Aleiodes *[57,58]. Moreover, according to Brues' (1933) description, some of the morphological features present in *R. fritschii *(e.g. ovipositor very short, antennae with only 25 segments, though most extant species have considerably more flagellomeres than this) appear to differ from the currently accepted concept of *Rogas*, but are more similar to the ones displayed by *Aleiodes sensu *van Achterberg (1991). Thus, it seems likely that the above specimen belongs to *Aleiodes sensu *van Achterberg [13] and not to *Rogas*. We therefore performed the PL and RP analyses including the calibration ages estimated for the Baltic amber to set the MRCAs of Clinocentrini and the *Aleiodes *clade (i.e. Aleiodini; see results), and including them to set the MRCA of Clinocentrini alone.

The age of the Baltic amber was originally proposed by Kaplan et al. [59] to be of 37.7 ± 3 MYA based on the absolute age of the Prussian Formation, though it was subsequently changed by Ritzowski [60], who inferred for this amber a Middle Eocene age (44.1 ± 1 MYA) based on radiometrically dated glauconite. The latter age estimate was employed in a recent phylogenetic analysis among the microgastroid genera [61]; however, it has been considered as doubtful by Perkovsky et al. [62] because it was calculated based on an insufficient layer sampling. We thus followed a conservative approach and set the MRCA of *Clinocentrus *Haliday to have a minimum age of 34.7 MYA in the PL analyses. Given the uncertainties associated with the fossil of *Aleiodes s. l.*, we did two separate analyses for the RP approach: including and excluding Aleiodini's calibration. We used the same prior distribution for the age of both Aleiodini and *Clinocentrus *MRCAs, an exponential distribution with a minimum age of 34.7 MYA and a 45.1 MYA upper bound (95% limit). We chose an exponential distribution with a zero offset of 34.7 to reflect our higher confidence in this value than in the 45.1 MYA upper bound.

### Evolution of host ranges and mummification

The evolution of lepidopteran host ranges and of two mummification features (position of emergence hole and degree of hardening of mummy) within the Rogadinae were assessed using MP and Bayesian ancestral reconstruction methods. Reconstruction of ancestral states with the MP method was performed mapping each character onto our preferred Bayesian phylogeny with MacClade version 4.0 [63]. Bayesian ancestral reconstructions of selected clades were carried with Bayestraits version 1.0 [64,65]; http://www.evolution.rdg.ac.uk), mapping each character onto a posterior distribution represented by the last 250 topologies sampled from the two independent searches (500 topologies in total) run for the simultaneous Bayesian analysis. Two searches of 10 million iterations each were run for each character, sampling a reconstruction of ancestral states every 500 iterations. We used a gamma-distributed hyperprior of the transition probabilities between states (rjhp exp 0.0 30) and a deviation rate of the normal distribution of 2.0. Burn-in was determined to be after 300,000 generations. The APP for the selected nodes were obtained by multiplying the mean ancestral character state probability of a given node across all trees by the portion of the trees in which the node involved was found [64,65].

**Figure 7:**
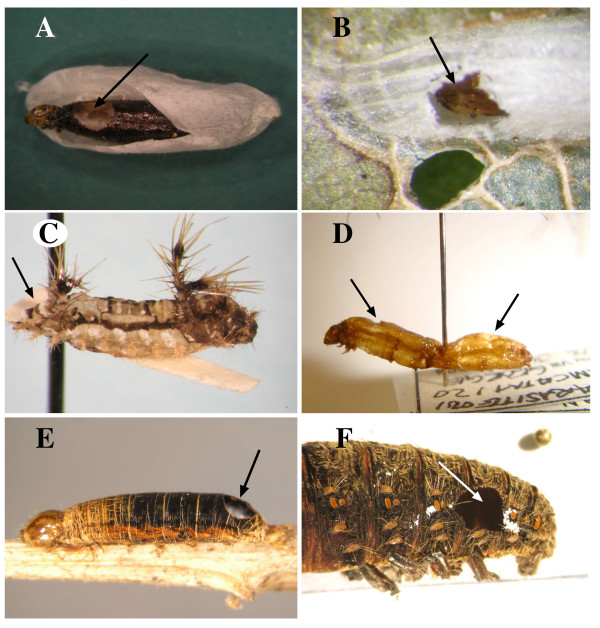
**Photographs of Rogadinae mummies**. Photographs showing the variety of lepidopteran mummy types among the currently recognised Rogadinae tribes. Arrows indicate the emergence holes. A. *Clinocentrus cunctator *(Haliday), ex *Anthophila fabriciana *(Linnaeus) (Choreutidae). B. *Stiropius bucculatricis *(Ashmead), ex *Bucculatrix ainsliella *Murtfeldt (Bucculatricidae). The mummy is inside the lepidopteran cocoon and is only visible through the exit hole. C. *Triraphis fusciceps *(Cresson), ex *Sibine *sp. (Limacodidae). D. *Colastomion *sp., ex Crambidae. E. *Aleiodes *(*Aleiodes*) sp. near *borealis *(Thomson), ex noctuid. F. *Cystomastax *sp., ex Arctiidae.

Ten states were defined for the host ranges character [(0) other insect groups, (1) Noctuidae, (2) other Noctuoidea, (3) Geometridae, (4) Rhopalocera, (5) Sphingidae, (6) Drepanidae + Thyratiridae, (7) Lasiocampidae, (8) Zygaenoidea, and (9) other microlepidopterans]. Four states were defined for the site of emergence [(0) not applicable because mummy absent, (1) anterior (Figures 7A, B), (2) posterior (Figures 7C,E), and (3) gregarious, various irregular positions (Figure 7D)] and four for the hardening of mummy [(0) absent, (1) weak (Figures 7C,D), (2) moderate, (3) hard (Figures 7E,F]. The host groups and mummification features recorded for the taxa examined are listed in Additional file 5. We only accepted the host record information that was confirmed after the direct examination of specimens and their host remains, though for a few taxa we accepted literature records in the absence of other evidence. In some cases, the host records given are those known for the species sequenced; however, host assignations for the remaining species (species of taxa listed only as genera in Appendix) were obtained from records of other species belonging to the same genus.

## Authors' contributions

AZR conceived of the study together with DLJQ, prepared the manuscript, generated part of the DNA sequences, carried out the sequence alignments, and performed the penalised likelihood and Bayesian phylogenetic analyses with MrBayes. MRS identified the *Aleiodes *species and part of the specimens from other genera, gathered and confirmed the host record information, contributed to the discussion of results, and revised the manuscript drafts. AGS generated part of the DNA sequences, carried out the BEAST analyses, performed part of the phylogenetic analyses, and revised the manuscript drafts. MM generated most of the DNA sequences. SAB selected and identified the fossil species and contributed to the taxonomic inferences and discussion of results. SRS contributed part of the host record information and to the discussion of results. DLJQ coordinated the study, identified part of the specimens, and revised the manuscript drafts. All authors helped to improve and approved the final manuscript.

## Supplementary Material

Additional file 1 Localities and voucher and EMBL/GenBank accession numbers of the species examined. Localities and voucher and EMBL/GenBank accession numbers of the species examined. Click here for file

Additional file 2 28S + COI matrix. DNA sequence matrix employed for the phylogenetic analyses performed for this study. Click here for file

Additional file 3 BEAST file excluding calibration of *Aleiodes s. l.*. input employed to perform the Bayesian relaxed phylogenetic analyses with the program BEAST (excluding calibration of *Aleiodes s. l*.) Click here for file

Additional file 4 BEAST file including calibration of *Aleiodes s. l.*. input employed to perform the Bayesian relaxed phylogenetic analyses with the program BEAST (including calibration of *Aleiodes s. l*.) Click here for file

Additional file 5 Host groups and mummy host features recorded for this study. Table mentioning the host groups and mummy host features recorded for this study. Click here for file
