# Supplementary material for: Evolution of the parasitic wasp subfamily Rogadinae (Braconidae): phylogeny and evolution of lepidopteran host ranges and mummy characteristics
Source: BMC Evol Biol. 2008 Dec 4;8:329. doi: 10.1186/1471-2148-8-329 (PMC2614994; doi:10.1186/1471-2148-8-329)
Supplement: Additional file 1 — Localities and voucher and EMBL/GenBank accession numbers of the species examined. Localities and voucher and EMBL/GenBank accession numbers of the species examined. [file 1471-2148-8-329-S1.doc]

**Table 1.** **Localities and voucher and EMBL/GenBank accession numbers of the species examined.**

| Taxon | Voucher no. | 28S | COI |
| --- | --- | --- | --- |
| **ROGADINAE** |  |  |  |
| **Clinocentrini** |  |  |  |
| *Anachyra* sp., Cameron Highlands, Malaysia ^b^ | – | AY935463* | – |
| *Artocella* *askewi* Shaw, Zaragoza, Spain ^d^ | – | AY935451* | AY935379* |
| *Clinocentrus* *cunctator* (Haliday), Ascot, Berkshire, UK ^b^ | Jo-702 | AJ784962* | AY935378* |
| *Tebennotoma* sp., Taiwan ^e^ |  | AJ784933* | AY935380* |
| **Aleiodini nov.** |  |  |  |
| ***Aleiodes*** |  |  |  |
| **Subgenus *Aleiodes*** |  |  |  |
| ***albitibia* group** |  |  |  |
| *Aleiodes albitibia* (Herrich-Schäffer), Ascot, Berkshire, UK ^d^ | MRS-58 | EU854323 | – |
| *Aleiodes albitibia*, Threepwood Moss, Roxburghshire, UK ^d^ | MRS-210 | – | EU979574 |
| ***circumscriptus* group** |  |  |  |
| *Aleiodes* sp. near *borealis* (Thomson), Ascot, Bershire | AL-120 | EU854326 | EU979578 |
| *Aleiodes circumscriptus* (Nees), Ascot, Berkshire, UK ^d^ | MRS-62 | EU854327 | EU979579 |
| *Aleiodes nigricornis* Wesmael, Loch Kinnordy, Angus, UK ^d^ | MRS-216 | AJ784934* | EU979585 |
| *Aleiodes pictus* (Herrich-Schäffer), Ascot, Berkshire, UK ^d^ | AL-119 | EU480587* | EF115464* |
| *Aleiodes similis* (Curtis), Ascot, Berkshire, UK ^d^ | AL-169 | EU854338 | EU979589 |
| ***compressor* group** |  |  |  |
| *Aleiodes compressor* (Herrich-Schäffer) [ex *Hydriomena furcata* (Thunberg)], Witherslack, Cumbria, UK ^d^ | MRS-170 | EU854328 | EU979580 |
| ***coxalis* group** |  |  |  |
| *Aleiodes coxalis* (Spinola), Ascot, Berkshire, UK ^d^ | MRS-80 | EU480588* | EF115460* |
| ***gastritor* group** |  |  |  |
| *Aleiodes* cf. *gastritor* (Thunberg)-agg. [ex *Operophtera brumata* (L.)], Aldclune, Perthshire, UK ^d^ | MRS-26 | EU854331 | EU979583 |
| *Aleiodes testaceus* (Telenga), Ascot, Berkshire, UK ^d^ | AL-058 | EF115439* | EF115454* |
| ***pallidator* group** |  |  |  |
| *Aleiodes pallidator* (Thunberg) [ex *Leucoma salicis* (L.)], Beynam, Ankara, Turkey ^d^ | MRS-1 | EU854333 | EU979586 |
| ***pilosus* group** |  |  |  |
| *Aleiodes* (*Tetrasphaeropyx*) sp., Sonora, Mexico ^c^ | – | EU854353 | – |
| ***praetor* group** |  |  |  |
| *Aleiodes* (*Neorhorgas*) praetor (Reinhard), Ascot, Berkshire, UK ^d^ | MRS-67 | EU854334 | – |
| ***seriatus* group** |  |  |  |
| *Aleiodes seriatus* (Herrich-Schäffer), St. Alvère, Dordogne, France ^d^ | MRS-136 | EU854337 | AY935368* |
| **“mummified hosts” group** |  |  |  |
| *Aleiodes barnardae* Quicke and Shaw [ex ennomine geometrid], Kibale, Uganda ^d^ | AL-324 | EF115431* | EF115446* |
| *Aleiodes mubfsi* Quicke and Shaw [ex ennomine geometrid], Kibale, Uganda ^d^ | AL-323 | EF115432* | EF115447* |
| *Aleiodes trevelyanae* Quicke and Shaw [ex ennomine geometrid], Kibale, Uganda ^d^ | AL-226 | EF115433* | EF115448* |
| *Aleiodes* sp. 7, Naivasha, Kenya ^d^ | AL-468 | EF115437* | EF111452* |
| **unplaced taxa** |  |  |  |
| *Aleiodes apiculatus* (Fahringer), Ascot, Berkshire, UK ^d^ | MRS-28 | EF115440* | EU979576 |
| *Aleiodes esenbeckii* (Hartig), Salla, Finland ^d^ | MRS-180 | EU854329 | EU979581 |
| *Aleiodes punctipes* (Thomson), Loch Kinnordy, Angus, UK ^d^ | MRS-212 | EU854335 | EU979587 |
| *Aleiodes* (*Cordylorhogas*) *trifasciatus* **n. comb.** Enderlein, Nyika N.P., N. Malawi ^c^ | Jo-874 | EU854354 | EU979604 |
| *Aleiodes* sp. 1, Madagascar ^d^ | AL-014 | EU854339 | EU979590 |
| *Aleiodes* sp. 2, Phuna Suna, Thailand ^d^ | AL-033 | EU854340 | EU979591 |
| *Aleiodes* sp. 3, Osaka, Japan ^d^ | AL-304 | EU854341 | EU979592 |
| *Aleiodes* sp. 4, Chillan, Chile ^d^ | AL-310 | EU854342 | EU979593 |
| *Aleiodes* sp. 5, Cameroun ^d^ | AL-045 | EU854343 | EU979594 |
| *Aleiodes* sp. 6, Cameron Highlands, W. Malaysia ^d^ | AL-079 | EU854344 | EU979595 |
| *Aleiodes* sp. 8, Abernethy, Inverness-shire, UK ^d^ | MRS-135 | EU854345 | EU979596 |
| *Aleiodes* sp. 9 [ex *Eupithecia pusillata* (Denis & Schiffermüller)], Inverness-shire, UK ^d^ | MRS-18 | EU854346 | EU979597 |
| *Aleiodes* sp. 11, Abedares Country Club, Mweiga, Kenya ^d^ | AL-451 | EU854348 | EU979599 |
| *Aleiodes* sp. 12, Mt. Kinabalu, Sabah, Indonesia ^d^ | AL-187 | EU854349 | EU979600 |
| *Aleiodes* sp. 13, Canberra, Australia ^d^ | AL-141 | EU854350 | EU979601 |
| *Aleiodes* sp. 15, Shizuoka, Atami, Japan ^d^ | AL-298 | EU854352 | EU979603 |
| **Subgenus *Arcaleiodes*** |  |  |  |
| *Arcaleiodes aglaurus* (Chen & He), China ^e^ | – | AY167657* | – |
| **Subgenus *Chelonorhogas*** |  |  |  |
| ***apicalis* group** |  |  |  |
| *Aleiodes apicalis* (Brullé), Beynam, Ankara, Turkey ^d^ | MRS-8 | EU854324 | EU979575 |
| ***gasterator* group** |  |  |  |
| *Aleiodes gasterator* (Jurine), Val de Restonica, Corsica, France ^d^ | MRS-46 | EU854330 | EU979582 |
| *Aleiodes grassator* (Thunberg) [ex *Tholera decimalis* (Poda)], Veszprém, Hungary ^d^ | MRS-163 | EU854332 | EU979584 |
| *Aleiodes ruficornis* (Herrich- Schäffer), Ascot, Berkshire, UK ^d^ | AL-144 | EU854336 | EF115477* |
| *Aleiodes unipunctator* (Thunberg), Loch Kinnordy, Angus, UK ^d^ | MRS-211 | EU480584* | EF115456* |
| ***melanopterus* group** |  |  |  |
| *Aleiodes aestuosus* (Reinhard), Sivas, Turkey ^d^ | MRS-4 | EU854322 | EU979573 |
| ***rugulosus* group** |  |  |  |
| *Aleiodes dissector* (Nees) [ex *Orthosia incerta* (Hufnagel)], Aldclune, Perthshire, UK ^d^ | MRS-146 | EU480585* | EF115471* |
| **unplaced taxa** |  |  |  |
| *Aleiodes aterrimus* (Ratzeburg) [ex *Amphipyra* sp.], Ascot, Berkshire, UK ^d^ | MRS-147 | EU854325 | EU979577 |
| *Aleiodes* (*Hemigyroneuron*) sp., Madagascar ^f^ | CAS 10481 | EU854375 |  |
| *Aleiodes* sp. 10, Sivas, Turkey ^d^ | MRS-126 | EU854347 | EU979598 |
| *Aleiodes* sp. 14, Costa Rica ^d^ | AL-490 | EU854351 | EU979602 |
| **Subgenus *Pholichora*** |  |  |  |
| *Aleiodes* (*Pholichora*) sp., Lapalala Wilderness, Transvaal, South Africa ^c^ | BF000887 | EU854383 | – |
| ***Heterogamus*** |  |  |  |
| *Heterogamus* *dispar* (Haliday), Ascot, Berkshire, UK ^d^ | AL-201 | AJ784935 | EU979605 |
| *Heterogamus* sp. 1, Australia ^d^ | AL-055 | EU854355 | EU979606 |
| *Heterogamus* sp. 2, Chiang Mae, Ban Mae Wan, Thailand ^d^ | AL-091 | EU854356 | EU979607 |
| *Heterogamus* sp. 3, Mt. Izumi Katsuragi, Japan ^d^ | AL-305 | EU854357 | EU979608 |
| *Heterogamus* *fasciatipennis* Ashmead, Duvberg, Härjedsdalen, Sweden ^d^ | MRS-394 | EU854358 | EU979609 |
| **Rogadini** |  |  |  |
| *Batothecoides yakushimensis*, Taiwan ^c^ | – | EU854360 | – |
| *Canalirogas* sp. 1, Cameron Highlands, W. Malaysia ^d^ | AL-190 | EU854361 | EU979610 |
| *Canalirogas* sp. 2, Phuna Suna, Thailand ^c^ | AL-112 | EU854362 | – |
| *Canalirogas* sp. 3, no data ^d^ | AL-165 | EU854363 | – |
| *Canalirogas* sp. 4, Sulawesi, Indonesia ^c^ | BF000633 | EU854364 | – |
| *Canalirogas* sp. 5, Sulawesi, Indonesia ^c^ | BF000640 | EU854365 | – |
| *Colastomion* *concolor* (Szépligeti) 1, Benin ^b^ | AL-125 | AY935446* | AY935370* |
| *Colastomion concolor* (Szépligeti) 2, Benin ^c^ | – | EU854366 | – |
| *Conspinaria* sp. 1, [ex *Chalosia thaivana owadai* Wang], Taitung Hsien Lanyu Island, Taiwan ^d^ | AL-198 | AJ509014* | EU480586* |
| *Conspinaria* sp. 2, [ex *Chalcosia diana* Butler], Taipei City, Taiwan ^c^ | AL-307 | EU854367 | EU979611 |
| *Conspinaria* sp. 3, [ex *Erasmia pulchella hobsoni* Butler], Taipei City, Taiwan ^d^ | AL-199 | AJ509015* | – |
| *Conspinaria* sp. 4, Chantaburi, Thailand ^d^ | AL-083 | EU854368 | EU979612 |
| *Conspinaria* sp. 5, Taiwan ^c^ | – | EU854369 | – |
| *Cornutorogas* *javensis* Achterberg, Mt. Halimun, Java, Indonesia ^c^ | – | EU854370 | – |
| *Cystomastax* sp. 1, Las Cuevas, Belize ^d^ | MRS-391 | EU854371 | EU979613 |
| *Cystomastax* sp. 2, Padrón, Caucagua, Venezuela ^b^ | Jo-574 | AY935445* | AY935369* |
| *Darnilia* sp. 1, Ninh Binh, Cuc Phuong N.P., N.Vietnam ^c^ | BF000917 | EU854403 | – |
| *Darnilia* sp. 2, Ninh Binh, Cuc Phuong N.P., N.Vietnam  ^c^ | BF000935 | EU854404 | – |
| *Gyroneuron* *testaceator* Watanabe, China ^c^ | MMF16 | EU854372 | EU979614 |
| *Gyroneuron* sp., Tun Yun, Taiwan ^c^ | AL-318 | EU854373 | – |
| *Gyroneuronella* sp., Gunung Buda, Sarawak, E. Malaysia (Borneo) ^c^ | Jo-802 | EU854374 | – |
| *Macrostomion* sp. 1, Chiang Dao, Chiang Mai, Thailand ^c^ | AL-110 | EU854376 | – |
| *Macrostomion* sp. 2, Taiwan ^c^ | BF002192 | EU854377 | – |
| *Megarhogas* *maculipennis* Chen & He, Chanta Bari, Pong Nani Ron, Thailand ^d^ | AL-138 | EU854379 | EU979615 |
| *Megarhogas* sp. No data ^c^ | – | EU854378 | – |
| *Myocron* sp. 1, Amani Hills, Tanzania ^d^ | AL-131 | EU854380 | EU979616 |
| *Myocron* sp. 2, Kibale Forest N. P., Uganda ^d^ | AL-216 | EU854381 | EU979617 |
| *Myocron* sp. 3, IITA compound, Ibadan, Nigeria ^c^ | BF000612 | EU854382 | – |
| *Rectivena* sp. 1, Benin ^d^ | AL-204 | EU854384 | EU979618 |
| *Rectivena* sp. 2, Kibale Forest N. P., Uganda ^c^ | AL-218 | EU854385 | EU979619 |
| *Rogas* sp., Amani Gate, Tanzania ^a^ | AL-170 | AJ784931* | AY935364* |
| *Rogasella* sp. 1, Negara, W. Bali, Indonesia ^c^ | BF000920 | EU854397 | – |
| *Rogasella* sp. 2, Thanh Son, Thuong Cuu, Vietnam ^c^ | BF000921 | EU854398 | – |
| *Spinaria albiventris* Cameron, Taiwan ^c^ | BF000801 | EU854401 | – |
| *Spinaria armator* ^1^ Fabricius, Sulawesi, Indonesia ^e^ | – | Z97964 | – |
| *Spinaria flavipennis,* Cameron, Taiwan ^c^ | F000802 | EU854402 | – |
| *Spinaria suliana* Westwood, Sulawesi ^c^ | BF000792 | EU854400 | – |
| *Spinaria* sp., Cameron Highlands, West Malaysia ^c^ | AL-173 | EU854399 | EU979623 |
| *Triraphis* sp. 1, Las Cuevas Belize ^c^ | AL-004 | EU854386 | EU979620 |
| *Triraphis* sp. 2, Kibale Forest N. P., Uganda ^c^ | AL-219 | EU854387 | EU979621 |
| *Triraphis* sp. 3, Costa Rica ^d^ | AL-493 | EU854388 | EU979623 |
| *Triraphis* sp. 4, Manu N. P., Madre de Dios, Peru ^c^ | AL-022 | EU854389 | – |
| *Triraphis* sp. 5, Manu N. P., Madre de Dios, Peru ^d^ | AL-062 | EU854390 | – |
| *Triraphis* sp. 6, Manu N. P., Madre de Dios, Peru ^c^ | AL-280 | EU854391 | – |
| *Triraphis* sp. 7, Las Cuevas, Belize ^d^ | AL-114 | EU854392 | – |
| *Triraphis* sp. 8, Las Cuevas, Belize ^c^ | BF000275 | EU854393 | – |
| *Triraphis* sp. 9, Manu N. P., Madre de Dios, Perú ^d^ | AL-064 | EU854394 | – |
| *Triraphis* sp. 10, Costa Rica ^d^ | AL-499 | EU854395 | – |
| *Triraphis* sp. 11, Kamarang, Guyana ^c^ | BF000698 | EU854396 | – |
| **Stiropiini** |  |  |  |
| *Choreborogas* sp., Costa Rica ^d^ | AL-508 | AY935447* | – |
| *Polystenidea* sp., Magdalena, Colombia ^b^ | Jo-821 | AY935448* | AY935374* |
| *Stiropius* sp., Costa Rica ^a^ | Jo-730 | AJ784961* | AY935373* |
| **Yeliconini** |  |  |  |
| *Asiabregma* sp., Malaysia ^c^ | – | AY935462* | – |
| *Bulborogas* sp. 1, Montagne de Kaw, French Guyana ^e^ | AL-203 | EU854359 | AY935372* |
| *Bulborogas* sp. 2, Caquetá, Chiribiqueta, Colombia ^c^ | AL-202 | AJ784930* | – |
| *Facitorus* sp., Hoa Binh, Mai Chau District, Vietnam ^a^ | Hym-011 | EU450765 | EU450766 |
| *Pseudoyelicones limonensis* Areekul & Quicke, Quepos Province, Costa Rica ^h^ | Jo-738 | AJ784929* | – |
| *Yelicones spectabile* Areekul & Quicke, Tollara Province, Madagascar ^f^ | Jo-734 | AJ784319* | AY935375* |
| *Yelicones nipponensis* Togashi, Taiwan ^c^ |  | AJ784324* | – |
| *Yelicones fischeri* Areekul & Quicke, Tollara Province, Madagascar ^f^ | Jo-735 | AJ784318* | – |
| *Yelicones zitanae* Areekul & Quicke, La Selva Biological Station, Heredia, Costa Rica ^g^ | AL-116 | AJ784325* | – |
| *Yelicones belokobylskiji*, Quicke, Chisti & Chen, Chon Buri, Khao Kheon, Thailand ^c^ | AL-124 | AJ784322* | – |
| **ALYSIINAE** |  |  |  |
| *Phaenocarpa* *ruficeps* (Nees), Ascot, Bershire, UK ^b^ | Jo-641 | AY935424* | AY935344* |
| **BETYLOBRACONINAE** |  |  |  |
| *Aulosaphobracon capitatus* Belokobylskij & Long*,* Mai Chau, Hoa Binh, Vietnam ^a^ | – | EU450764 | – |
| *Betylobracon* *waterhousi* Tobias, Australia ^e^ | – | AJ245686* | – |
| *Mesocentrus* sp., Canberra, Australia ^b^ | Jo-645 | AY935461* | – |
| **BRACONINAE** |  |  |  |
| *Bracon* sp., Kibale Forest N. P., Uganda ^b^ | Jo-775 | AY935436* | AY935353* |
| *Pseudoshirakia* sp., South Korea ^c^ | – | AY935438* | AY935359* |
| *Megacoeloides* sp., Columbia ^c^ | – | AY935939* | AY935360* |
| *Spinadesha* sp., Malaysia ^c^ | – | AY935440* | AY935361* |
| **DORYCTINAE** |  |  |  |
| *Aivalykus* *arawak* Marsh, Costa Rica ^a^ | Jo-725 | AY935471* | AY935398* |
| *Aivalykus cornicoxa* (Braet & Barbalho), French Guyana (holotype) ^b^ | Jo-840 | EF645757* | EF645790* |
| *Aivalykus* sp., Costa Rica ^a^ | Jo-741 | EF645758* | EF645791* |
| *Stenocorse* sp., Belize ^a^ | Jo-750 | AY935475* | AY935402* |
| *Heterospilus prosopidis* Viereck, ex Silwood culture, UK ^e^ | Jo-601 | AY935469* | AY935396* |
| **EXOTHECINAE** |  |  |  |
| *Colastes* sp., Ascot, Berkshire, UK ^b^ | Jo-698 | AY935431* | AY935350* |
| *Xenarcha abnormis* (Wesmael), Primorski Krai, Russia ^a^ | Jo-883 | AY935434* | AY935352* |
| **GNAMPTODONTINAE** |  |  |  |
| *Gnaptogaster astrachanica* Belokobylskij, Astrakhan’ Province, Russia ^a^ | Jo-894 | AY935441* | AY935363* |
| **HORMIINAE** |  |  |  |
| *Hormius* sp., Mahajanga Province, Madagascar ^b^ | Jo-582 | AY935455* | AY935385* |
| *Parahormius* sp., Mt. Coupe, Cameroon ^a^ | Jo-576 | AY935456* | AY935386* |
| *Pentatermus* sp., Benin ^a^ | Jo-695 | AY935453* | AY935383* |
| **LYSITERMINAE** |  |  |  |
| *Acanthormius* sp., Madagascar ^a^ | Jo-692 | AJ302883* | AY935381* |
| *Atritermus pedestris* Belokobylskij, Zaldivar-Riverón & Quicke, Madagascar (paratype) ^a^ | – | DQ414401* | – |
| *Carinitermus* *reticulatus*, Kibale Forest N. P., Uganda ^c^ |  |  |  |
| *Cedria* sp., Madagascar ^a^ | Jo-579 | AY935460* | AY935390* |
| *Katytermus* sp., Honshu Hyogo, Kobe, Rokko Mts., Japan ^a^ | Hym-08 | EU854406 | EU979624 |
| *Lysitermus*, sp., Kibale Forest N. P., Uganda ^a^ | AL-220 | EU854405 | – |
| *Platyrmus maichaui*, Lac Thinh Cuc Phuong N. P., Hoa Binh, Vietnam ^a^ | Hym-12 | EU854407 | – |
| *Tetratermus* sp., Kibale Forest N. P., Uganda ^a^ | Jo-703 | AY935452* | AY935382* |
| **PAMBOLINAE** |  |  |  |
| *Pambolus* sp., Choroni, Venezuela ^b^ | Jo-597 | AY935458* | AY935388* |
| *Pseudorhysipolis* sp., Costa Rica ^b^ | Jo-758 | AY935450* | AY935377* |
| *Notiopambolus depressicauda* Achterberg & Quicke, Canberra, Australia ^b^ | Jo-651 | AY935459* | AY935389* |
| **RHYSSALINAE** |  |  |  |
| *Oncophanes* sp., Ascot, Berkshire, UK ^e^ | – | AY935481* | AY935407* |
| *Rhyssalus* *clavator* Haliday, Kazimierz, Poland ^a^ | Jo-890 | AY935482* | AY935409* |
| **RHYSIPOLINAE** |  |  |  |
| *Noserus flavicoxa* (Tobias), Russia ^b^ | Jo-696 | AY935454* | AY935384* |
| *Rhysipolis temporalis* Belokobylskij, Primorskii Krai, Russia ^a^ | Jo-886 | AY935449* | AY935376* |
| *Parachremylus* sp., Nigeria ^c^ | BF000619 | EU854408 | EU979625 |
| **MESOSTOINAE** |  |  |  |
| *Andesipolis* sp., Flor de Lago, Chile ^a^ | Jo-753 | AY935485* | AY935411* |
| *Aspilodemon* sp., Colombia ^a^ | Jo- 688 | AY935487* | AY935413* |
| *Mesostoa kerri* Austin & Wharton, Australia ^b^ | Jo-664 | AJ302930* | AY935415* |
| **UNPLACED GENERA** |  |  |  |
| *Allobracon* sp., Brazil ^e^ | – | AJ302886* | AY935391* |

The given classification within Rogadinae presented is based on the results obtained in this study. *Aleiodes* species are grouped into the species-groups proposed by Fortier & Shaw (1999). * = Sequences obtained from previous works. Voucher specimens: ^a^ = Zoological Institute, St. Petersburg, Russia; ^b^ = Nationaal Natuurhistorisch Museum, Leiden, Netherlands; ^c^  = Natural History Museum, London, UK; ^d^ = National Museums of Scotland, Edinburgh, Scotland; ^e^ = not retained/unknown; ^f^ = California Academy of Sciences, San Francisco, CA; ^g^ = University of Wyoming, Laramie, WY, USA; ^h^ = Instituto Nacional de Biodiversidad (INBio), Santo Domingo de Heredia, Costa Rica. ^1^ On GenBank as *S. fuscipennis* Brullé; this species was synonymised with *S. armator* Fabricius by van Achterberg (2007).

= Instituto Nacional de Biodiversidad (INBio), Santo Domingo de Heredia, Costa Rica. X = TO BE SUBMITTED.

|  |  |  |  |
| --- | --- | --- | --- |
|  |  |  |  |
|  |  |  |  |
|  |  |  |  |
|  |  |  |  |
|  |  |  |  |
|  |  |  |  |
|  |  |  |  |
|  |  |  |  |
|  |  |  |  |
|  |  |  |  |
|  |  |  |  |
|  |  |  |  |
|  |  |  |  |
|  |  |  |  |
|  |  |  |  |
|  |  |  |  |
|  |  |  |  |
|  |  |  |  |
|  |  |  |  |
|  |  |  |  |
|  |  |  |  |
|  |  |  |  |
|  |  |  |  |
|  |  |  |  |
|  |  |  |  |
|  |  |  |  |
|  |  |  |  |
|  |  |  |  |
|  |  |  |  |
|  |  |  |  |
|  |  |  |  |
|  |  |  |  |
|  |  |  |  |
|  |  |  |  |
|  |  |  |  |
|  |  |  |  |
|  |  |  |  |
|  |  |  |  |
|  |  |  |  |
|  |  |  |  |
|  |  |  |  |
|  |  |  |  |
|  |  |  |  |
|  |  |  |  |
|  |  |  |  |
|  |  |  |  |
|  |  |  |  |
|  |  |  |  |
|  |  |  |  |
|  |  |  |  |
|  |  |  |  |
|  |  |  |  |
|  |  |  |  |
|  |  |  |  |
|  |  |  |  |
|  |  |  |  |
|  |  |  |  |
|  |  |  |  |
|  |  |  |  |
|  |  |  |  |
|  |  |  |  |
|  |  |  |  |
|  |  |  |  |
|  |  |  |  |
|  |  |  |  |
|  |  |  |  |
|  |  |  |  |
|  |  |  |  |
|  |  |  |  |
|  |  |  |  |
|  |  |  |  |
|  |  |  |  |
|  |  |  |  |
|  |  |  |  |
|  |  |  |  |
|  |  |  |  |
|  |  |  |  |
|  |  |  |  |
|  |  |  |  |
|  |  |  |  |
|  |  |  |  |
|  |  |  |  |
|  |  |  |  |
|  |  |  |  |
|  |  |  |  |
|  |  |  |  |
|  |  |  |  |
|  |  |  |  |
|  |  |  |  |
|  |  |  |  |
|  |  |  |  |
|  |  |  |  |
|  |  |  |  |
|  |  |  |  |
|  |  |  |  |
|  |  |  |  |
|  |  |  |  |
|  |  |  |  |
|  |  |  |  |
|  |  |  |  |
|  |  |  |  |
|  |  |  |  |
|  |  |  |  |
|  |  |  |  |
|  |  |  |  |
|  |  |  |  |
|  |  |  |  |
|  |  |  |  |
|  |  |  |  |
|  |  |  |  |
|  |  |  |  |
|  |  |  |  |
|  |  |  |  |
|  |  |  |  |
|  |  |  |  |
|  |  |  |  |
|  |  |  |  |
|  |  |  |  |
|  |  |  |  |
|  |  |  |  |
|  |  |  |  |
|  |  |  |  |
|  |  |  |  |
|  |  |  |  |
|  |  |  |  |
|  |  |  |  |
|  |  |  |  |
|  |  |  |  |
|  |  |  |  |
|  |  |  |  |
|  |  |  |  |
|  |  |  |  |
|  |  |  |  |
|  |  |  |  |
|  |  |  |  |
|  |  |  |  |
|  |  |  |  |
|  |  |  |  |
|  |  |  |  |
|  |  |  |  |
|  |  |  |  |
|  |  |  |  |
|  |  |  |  |
|  |  |  |  |
|  |  |  |  |
|  |  |  |  |
|  |  |  |  |
|  |  |  |  |
|  |  |  |  |
|  |  |  |  |
|  |  |  |  |
|  |  |  |  |
|  |  |  |  |
|  |  |  |  |
|  |  |  |  |
|  |  |  |  |
|  |  |  |  |
|  |  |  |  |
|  |  |  |  |
|  |  |  |  |
|  |  |  |  |
|  |  |  |  |
|  |  |  |  |
|  |  |  |  |
|  |  |  |  |
|  |  |  |  |
|  |  |  |  |
|  |  |  |  |
|  |  |  |  |
|  |  |  |  |
|  |  |  |  |
|  |  |  |  |
|  |  |  |  |
|  |  |  |  |
|  |  |  |  |
|  |  |  |  |
|  |  |  |  |
|  |  |  |  |
|  |  |  |  |
|  |  |  |  |
|  |  |  |  |
|  |  |  |  |
|  |  |  |  |
|  |  |  |  |
|  |  |  |  |
|  |  |  |  |
|  |  |  |  |
|  |  |  |  |
|  |  |  |  |
|  |  |  |  |
|  |  |  |  |
|  |  |  |  |
|  |  |  |  |
|  |  |  |  |
|  |  |  |  |
|  |  |  |  |
|  |  |  |  |
|  |  |  |  |
|  |  |  |  |
