# Supplementary material for: Evolution of the parasitic wasp subfamily Rogadinae (Braconidae): phylogeny and evolution of lepidopteran host ranges and mummy characteristics
Source: BMC Evol Biol. 2008 Dec 4;8:329. doi: 10.1186/1471-2148-8-329 (PMC2614994; doi:10.1186/1471-2148-8-329)
Supplement: Additional file 5 — Host groups and mummy host features recorded for this study. Table mentioning the host groups and mummy host features recorded for this study. [file 1471-2148-8-329-S5.doc]

**Additional file 5.** **Host groups and mummy host features recorded for this study.**

| Taxon | Host range type ^1^ | Lepidopteran host | Site of emergence from mummy | Hardening of mummy | References |
| --- | --- | --- | --- | --- | --- |
| Aleiodini **tribe** **nov.** |  |  |  |  |  |
| *Aleiodes aestuosus* | ? | Noctuidae (*Heliothis****) | Posterodorsal | Hard |  |
| *Aleiodes albitibia* | C | Notodontidae (*Notodonta**, *Eligmodonta**, *Pheosia**) | Posterodorsal | Hard | [1] |
| *Aleiodes apicalis* | C | Noctuidae (*Autographa**) | Posterodorsal | Hard |  |
| *Aleiodes apiculatus* | C | Lymantriidae (*Euproctis**) | Posterodorsal | Hard |  |
| *Aleiodes aterrimus* | C | Noctuidae (*Amphipyra**) | Posterodorsal | Hard |  |
| *Aleiodes barnardae* | C? | Geometridae: Ennominae | Posterodorsal | Hard | [2] |
| *Aleiodes* near *borealis* | D | Noctuidae (*Noctua**, *Xestia**, *Stilbia**, *Phlogophora**, *Euplexia**, *Cucullia**) | Posterodorsal | Hard |  |
| *Aleiodes circumscriptus* | C | Noctuidae (*Hypena**) | Posterodorsal | Hard |  |
| *Aleiodes compressor* | D | Geometridae (*Hydriomena**); Notodontidae (*Clostera**); Thyratiridae (*Achlya**); Noctuidae (indet.*) | Posterodorsal | Hard |  |
| *Aleidoes coxalis* | D | Satyridae (*Maniola**, *Melanargia**, *Coenonympha**), Hesperiidae (*Thymelicus**) | Posterodorsal | Hard |  |
| *Aleiodes dissector* | C | Noctuidae (*Orthosia**) | Posterodorsal | Hard |  |
| *Aleiodes dispar* group (*Heterogamus*) | ? | Unknown ^2^ |  |  |  |
| *Aleiodes esenbeckii* | C | Lasiocampidae (*Dendrolimus**) | Posterodorsal | Hard |  |
| *Aleiodes gasterator* | C | Noctuidae (*Agrotis**) | Posterodorsal | Hard |  |
| *Aleiodes* cf. *gastritor* | D | Geometridae (*Operophtera**, *Alsophila**); Noctuidae (*Conistra**) | Posterodorsal | Hard |  |
| *Aleiodes grassator* | C | Noctuidae: (*Cerapteryx**, *Tholera**) | Posterodorsal | Hard |  |
| *Aleiodes mubfsi* | C | Geometridae: Ennominae | Posterodorsal | Hard | [2] |
| *Aleiodes nigricornis* | D | Noctuidae (*Orthosia**, *Apamea**) | Posterodorsal | Hard | [1] |
| *Aleiodes pallidator* | C | Lymantriidae (*Leucoma**) | Posterodorsal | Hard | [3] |
| *Aleiodes pictus* | D | Noctuidae (*Xestia**, *Diarsia**); Geometridae (*Camptogramma**, *Xanthorhoe**, *Lithostege**) | Posterodorsal | Hard |  |
| *Aleiodes praetor* | C | Sphingidae (*Mimas**, *Lathoe**) | Posterodorsal | Hard |  |
| *Aleiodes ruficornis* | D | Noctuidae (*Agrotis**, *Hoplodrina**) | Posterodorsal | Hard |  |
| *Aleiodes seriatus* | C | Arctiidae (*Atolmis**) | Posterodorsal | Hard |  |
| *Aleiodes similis* | D | Noctuidae (*Xestia**, *Orthosia**, *Conistra**) | Posterodorsal | Hard |  |
| *Aleiodes testaceus* | C | Geometridae (*Eupithecia**, *Chloroclystis**, *Gymnoscelis**) | Posterodorsal | Moderately hard |  |
| *Aleiodes trevelyanae* | C | Geometridae: Ennominae | Posterodorsal | Hard | [2] |
| *Aleiodes unipunctator* | C | Noctuidae (*Apamea**) | Posterodorsal | Hard |  |
| *Aleiodes* sp. (*pillosus* group) | C | Geometridae: Ennominae | Posterodorsal | Hard | [4] |
| *Aleiodes* sp. 9 | C | Geometridae (*Eupithecia**) | Posterodorsal | Hard |  |
| Clinocentrini |  |  |  |  |  |
| *Clinocentrus* | C ^3^ | Choreutidae*, Momphidae*, Ypsolophidae*, Epermeniidae*, Tortricidae* | Anterior, various radial orientations | Moderate to hard | [5] |
| Rogadini |  |  |  |  |  |
| *Canalirogas* | ? | Lymantriidae | Posterodorsal | Moderate | [5] |
| *Colastomion* | ?C | Crambidae | Gregarious, various irregular positions | Weak | This study |
| *Conspinaria* | ?C | Zygaenidae: Chalcosiinae | Posterior, various orientations | Moderate | [5] |
| *Cystomastax* | ? | Arctiidae** | Posterodorsal, posterolateral | Hard |  |
| *Darnilia* | ? | Limacodidae (*Darna*) | Unknown | Unknown | [6] |
| *Hemigyroneuron* | ? | Geometridae, Lasiocampidae | Posterodorsal | Hard | [5] |
| *Macrostomion* | ?C | Sphingidae | Gregarious, various irregular positions | Moderate | [7] [8] |
| *Megarhogas* | ? | Lymantriidae (*Perina*) | Posterolateral | Weak | [9] |
| *Pholichora* | ? | Geometridae: (*Ascotis*) | Unknown | Unknown | [10] |
| *Rectivena* | ? | Limacodidae | Posterodorsal, posterodorsolateral | Weak | [11] |
| *Rogas* | ? | Limacodidae (*Apoda**) | Anterodorsal ^4^ | Weak | M. R. Shaw (unpublished data), [5] |
| *Spinaria*, 9 species | C | Limacodidae | Posterodorsal, posterodorsolateral | Weak | [12] [13] |
| Old World *Triraphis* |  | Limacodidae (*Apoda**); Zygaenidae | Posterodorsal, posterodorsolateral | Weak/hard | [5], M. R. Shaw (unpublished data) |
| New World *Triraphis* |  | Zygaenidae (*Harrisina*); Limacodidae (*Sibine***, *Vipsania***, *Perola***); Dalceridae (*Acraga***); Megalopygidae (*Norape***); Riodinidae (*Melanis***, *Napaea***, *Emesis*); Lycaenidae (*Ocaria***, *Arawakis***, *Strymon**) | Posterodorsal, dorsolateral; sometimes gregarious, species not seen | Moderate | Valerio and Shaw (in prep.) |
| Stiropiini |  |  |  |  |  |
| *Choreborogas* |  | Lyonetiidae | Unknown | Unknown | [14] |
| *Polystenidea* |  | Bucculatrigidae | Unknown | Unknown | [14] |
| *Stiropius* |  | Bucculatrigidae, Lyonetiidae | Anterodorsal | Weak | [15] [5] |
| Yeliconini |  |  |  |  |  |
| *Yelicones* |  | Pyralidae | Posterodorsal, anterodorsal | Moderately weak | [16] |
| Other subfamilies |  |  |  |  |  |
| *Acanthormius* |  | Xyloryctidae |  |  | [17] |
| *Hormius* |  | Pyralidae, Coleophoridae***, Gelechiidae***, Momphidae***, Oecophoridae***, Tortricidae*** |  |  | [17] |
| *Lysitermus* |  | Psychidae |  |  | [17] |
| *Noserus* |  | Bucculatrigidae, Lyonetiidae*** |  |  | [14] |
| *Oncophanes* |  | Various microlepidopteran families |  |  | [14] |
| *Parahormius* |  | Lyonetiidae, Cosmopterigidae |  |  | [14] |
| *Pentatermus* |  | Noctuidae, Hesperidae*** |  |  | [11] |
| *Rhysipolis* |  | Gracillaridae, Momphidae |  |  | [14] |

* = Host records confirmed by MRS (unpublished primary data); ** = host records confirmed by SRS; *** = host record confirmed by SAB.

^1^ "C" means taxonomically continuous (contiguous); "D" means taxonomically discontinuous (i.e. unrelated) (see Shaw 2002)

^2^ Hosts listed in Fortier & Shaw (1999) appear to be derived from sources listed in Shenefelt (1975) dating from publications before 1930 with uncertain identifications; we have not seen any direct evidence that any member of the *A. dispar* group has ever been reared.

^3^ Each of the six species for which host range is known have a continuous host range.

^4^ For the type species *R.luteus* (Nees) eleven anterodorsal and only one posterodorsal emergences seen; the latter discounted as abnormal.

**REFERENCES**

1. Shaw MR: **Host ranges of *Aleiodes* species (Hymenoptera: Braconidae), and an evolutionary hypothesis**. In: *Parasitic wasps: evolution, systematics, biodiversity and biological control.* Edited by Melika G, Thuróczy C. Budapest: Agroinform; 2002: 322–327.

2. Quicke DLJ, Mori M, Zaldivar-Riverón A, Shaw MR: **Suspended mummies in *Aleiodes* species (Hymenoptera: Braconidae: Rogadinae) with descriptions of six new species form western Uganda based largely on DNA sequence data**. *J Nat Hist* 2006, **40**:2663–2680.

3. Dowden PB: ***Rogas unicolor* (Wesmael), a braconid parasite of the satin moth**. *J Agric Res* 1938, **56**:523–535.

4. Fortier JC: **Twelve new species and a new combination of the *Aleiodes pilosus* species-group (= *Tetrasphaeropyx* Ashmead) (Hymenoptera, Braconidae, Rogadinae) in North America: Part 1**. *Entomol News* 2006, **117**:465–484.

5. Quicke DLJ, Shaw MR: **First host records for the rogadine genera *Rogasodes* Chen and He and *Canalirogas* van Achterberg and Chen (Hymenoptera: Braconidae) with description of a new species and survey of mummy types within Rogadinae *s. str***. *J Nat Hist* 2005, **39**:3525–3542.

6. Achterberg C van: **Four new genera of Braconinae and Rogadinae from the Oriental region (Hymenoptera: Braconidae)**. *Zool Med* 1989, **63**:79–95.

7. Shaw MR: **A new species of *Macrostomion* Szépligeti (Hymenoptera: Braconidae: Rogadinae) from Papua New Guinea, with notes on the biology of the genus**. *Zool Med* 2002, **76**:133–140.

8. Maeto K, Arakaki N: **Gregarious emergence of *Macrostomion sumatranum* (Hymenoptera: Braconidae; Rogadinae) from the mummified, full-grown larvae of Theretra silhetensis (Lepidoptera: Sphingidae)**. *Entomol Sci* 2005, **8**:131–132.

9. Watanabe C: **Descriptions of new species of genera *Megarhogas*, *Cystomastax* and *Stantonia* (Braconidae) from Formosa**. *Insecta Matsum* 1932, **6**:184–189.

10. Quicke DLJ, Shaw MR: **First hot record for the rogadine genus *Pholichora* van Achterberg (Hymenoptera: Braconidae) with description of a new species and notes on convergent wing venation features**. *J Nat Hist* 2005, **39**:531–537.

11. Achterberg C van: **Revision of the Afrotropical genera and W. Palaearctical Rogadinae Foerster (Hymenoptera: Braconidae)**. *Zool Verh* 1991, **273**:1–102.

12. Austin AD: **Braconidae: a review of the Braconidae (Hymenoptera) that parasitize Limacodidae in Southeast Asia, particularly those associated with coconut and oil palm**. In: *The biology and control of the Limacodidae of economic importance on palms in SE Asia.* Edited by Cock MJW, Godfray HCJ, Holloway JD. Wallingford: CAB International; 1987: 139–164.

13. Achterberg C van: **Revision of the genus *Spinaria* Brullé (Hymenoptera: Braconidae: Rogadinae), with keys to genera and species of the subtribe Spinariina van Achterberg**. *Zool Med* 2007, **81**:11–83.

14. Whitfield JB, Wagner DL: **Annotated key to the genera of Braconidae (Hymenoptera) attacking leafmining Lepidoptera in the Holarctic region**. *J Nat Hist* 1991, **25**:733–754.

15. Whitfield JB: **Revision of the Nearctic species of the genus *Stiropius* Cameron (= *Bucculatriplex* Auct.) with the description of a new related genus (Hymenoptera: Braconidae)**. *Syst Entomol* 1988, **13**:373–385.

16. Quicke DLJ, Kruft RA: **Species of Yelicones (Hymenoptera: Braconidae: Rogadinae) in North America with descriptions of two new species**. *Ann Entomol Soc Am* 1995, **88**:129–138.

17. Achterberg C van: **Generic revision of the subfamily Betylobraconinae (Hymenoptera: Braconidae) and other groups with modified fore tarsus**. *Zool Verh* 1995, **298**:1–242.
